# Supplementary material for: A self-regulated photothermal anti-/deicing film for all-season applications
Source: Nat Commun. 2026 Feb 11;17:2632. doi: 10.1038/s41467-026-69494-x (PMC13004981; doi:10.1038/s41467-026-69494-x)
Supplement: Supplementary file 1 — Supplementary Information [file 41467_2026_69494_MOESM1_ESM.pdf]

Supplementary Information

A Self-Regulated Photothermal Anti-/Deicing Film for All-Season Applications

Jiayu Du[1], Wenqi Wang[1], Yang Fu[1], Xin Li[1], Jie Tan[1], Hao Li[1], Xu Chen[1], Fuqiang Chu[2,*], Qi Min[3], Chi Yan Tso[1,*]

1 School of Energy and Environment, City University of Hong Kong, Tat Chee Avenue, Kowloon Tong, Hong Kong, China

2 School of Energy and Environmental Engineering, University of Science and Technology Beijing, Beijing 100083, China

3 Key Laboratory of Advanced Reactor Engineering and Safety of Ministry of Education, Collaborative Innovation Center of Advanced Nuclear Energy Technology, Institute of Nuclear and New Energy Technology, Tsinghua University, Beijing 100084, China

*Corresponding authors.

Email: chiytso@cityu.edu.hk (C.Y.T.), chufq@ustb.edu.cn (F.C.)

This PDF file includes:

Supplementary Methods

Supplementary Figures 1 to 42

Supplementary Tables 1 to 7

Supplementary Notes 1 to 3

Supplementary References

Other supplementary information for this manuscript includes:

Supplementary Movies 1 to 12

Supplementary Methods

Surface wettability and durability tests

The contact angle (CA) and sliding angle (SA) were measured by gently depositing 10 μL deionized (DI) water droplets onto a horizontal and inclined surface, respectively. The sliding angle corresponds to the tilt angle of that initiates droplet sliding. The values of CA and SA were determined from five different regions on each surface. The static and dynamic behavior of droplets was captured using a high-speed camera (Phantom, Micro C110) at 1200 fps.

(1) Sandpaper abrasion test. The sample was placed face-down to a 1000 grit sandpaper, and then horizontally moved along the sandpaper under a 100 g weight (2.6 kPa). In each cycle, the abrasion distance was 20 cm.

(2) Tape-peeling test (ASTM D3359). The 3M tape (20 mm in width and 0.6 mm in thickness) with high adhesion strength of 3000 N/m to standard stainless steel was used. The tape was pressed onto the sample surface using a cylindrical copper block (11 kPa), which was peeled off after several seconds. After certain cycles, the CA and SA were measured. The mechanical durability of MNTS film was compared to that of a commercial agent (Glaco Mirror Coat "Zero"), an alcohol-based suspension of silica nanoparticles.

(3) Acid rain impact test. We used a shower with a nozzle diameter of 0.4 mm to form simulated acid rainfall (pH=5.0) with a flow of ~20 mL/s. The 15° tilted sample was placed 30 cm beneath the shower. The CA and SA were measured after certain time intervals.

(4) Sand impact test (ASTM D968). The silica sand (200-300 μm) fell freely from a height of 15 cm to impact the surface at a tilt angle of 45°. The CA and SA were measured after falling of certain mass of sand particles.

(5) UV resistance test. Accelerated UV aging test was conducted in a UV weathering chamber maintained at 25 °C (340 nm maximum intensity, UV irradiance of 0.89 W/m^2) for 4 weeks. This UV dosage is equivalent to 8 months of Florida sunshine exposure (annual UV dosage of about 275 $MJ \cdot m^{-2}$). Florida sunshine exposure is an international benchmark for durability tests of materials. The CA and SA were measured after every 4 days.

(6) Chemical stability test. The sample was immersed into tetradecane, 1 mol/L NaCl solution, HCl solution (pH=2) and NaOH solution (pH=12), respectively. The CA and SA of rinsed surface were measured after certain time intervals.

Optical performance characterization

The spectra in the solar range (300-2500 nm) were measured by a UV-Vis-NIR spectrophotometer (Lambda 1050, Perkin Elmer) equipped with a 150 mm integrating sphere. A ring-shaped ceramic heating plate (ϕ48-20 mm, 5 V, 9 W) was attached to the samples to measure the spectra at different temperatures. The solar transmittance (τ_{sol}) and reflectivity (R_{sol}) were calculated as follows

$$\tau_{sol} = \frac{\int_{300nm}^{2500nm} \mathrm{AM}_{1.5}(\lambda)\tau(\lambda)d\lambda}{\int_{300nm}^{2500nm} \mathrm{AM}_{1.5}(\lambda)d\lambda}, \quad (S1)$$

$$R_{sol} = \frac{\int_{300nm}^{2500nm} \mathrm{AM}_{1.5}(\lambda)R(\lambda)d\lambda}{\int_{300nm}^{2500nm} \mathrm{AM}_{1.5}(\lambda)d\lambda}, \quad (S2)$$

where $\tau(\lambda)$ and $R(\lambda)$ represent the transmittance and reflectivity at the wavelength of λ, respectively. $\mathrm{AM}_{1.5}(\lambda)$ represents the solar irradiance spectrum for air mass 1.5 (corresponding to the sun standing 37° above the horizon with 1.5-atm thickness, corresponding to a solar zenith angle of 48.2°). The absorptance spectrum in the solar range can be determined by $\alpha(\lambda)=1-\tau(\lambda)-R(\lambda)$. The transmittance modulation ($\Delta\tau_{sol}$) and reflectivity modulation (ΔR_{sol}) of thermochromic hydrogels were calculated by $\Delta\tau_{sol}=\tau_{sol,20°C}-\tau_{sol,40°C}$ and $\Delta R_{sol}=R_{sol,40°C}-R_{sol,20°C}$, respectively. Following ASTM D1003 "Standard Method for Haze and Luminous Transmittance of Transparent Plastics", the haze was calculated by $(\tau_4/\tau_2-\tau_3/\tau_1)\times100\%$, where τ_1 is the incident light, τ_2 is the total light transmitted by the sample, τ_3 is the light scattered by the equipment, and τ_4 is the light scattered by the sample and equipment.

The reflectivity and transmittance spectra in the wavelength ranging from 2.5 μm to 16 μm was measured via a Fourier transform infrared (FTIR) spectroscopy (IRAffinity-1S, Shumadzu) coupled with an integrating sphere (MID-IR IntegratIR, PIKE). The infrared emissivity in the atmospheric transmission window was calculated by

$$\varepsilon_{8\text{-}13} = \frac{\int_{8\mu m}^{13\mu m} I_{bb}(\lambda,T)\varepsilon(\lambda,T)d\lambda}{\int_{8\mu m}^{13\mu m} I_{bb}(\lambda,T)d\lambda}, \quad (S3)$$

where $I_{bb}(\lambda,T)$ is the spectral intensity for blackbody radiation at a temperature of T calculated by Plank's law, and $\varepsilon(\lambda,T)$ is the emissivity measured by FTIR spectrometer with an integrated sphere. The refractive index of samples was measured using an ellipsometer (Horoba UVISEL PLUS).

To measure the transition temperature of hydrogels, heating and cooling cycles were conducted,

where the sample temperatures were adjusted between 20 °C and 40 °C at certain intervals. The samples were kept on the heating plate for 5 min to maintain stability under each temperature set point. Subsequently, the transmittance spectrum was measured by the UV-Vis-NIR spectrophotometer. The transition temperature corresponded to the minimum value point of the first derivative of transmittance with respect to the temperature. To examine the stability of hydrogels after numerous heating/cooling cycles, the solar transmittance was measured at intervals of 10 times for a total of 100 cycles. The response time of hydrogels was determined by alternately placing the sample into constant-temperature water baths at 20 °C and 40 °C.

Phase change properties

The phase change properties were characterized using a differential scanning calorimeter (DSC25, TA instruments). For lower critical solution temperature (LCST) measurements, hydrogel samples were equilibrated at 10 °C for 2 min, followed by heating to 50 °C and cooling back to 10 °C. For freezing point measurements, samples were equilibrated at 10 °C for 2 min, followed by cooling to –40 °C. To evaluate the anti-freezing property, hydrogel samples were placed in a refrigerator at –20 °C for 3 h. To measure the phase change temperature and enthalpy of composite phase-change material (CPCM), samples were equilibrated at 15 °C for 2 min, followed by cooling to –30 °C and heating back to 15 °C. During all DSC tests, the heat and cooling cycles were conducted under a nitrogen atmosphere at a rate of 3 °C/min. The encapsulation efficiency (E_{en}), latent heat storage efficiency (E_{es}) and thermal storage capacity (C_{es}) of CPCM were calculated through the following equations:

$$E_{en} = \frac{\Delta H_{m,CPCM}}{\Delta H_{m,PCM}} \times 100\%, \tag{S4}$$

$$E_{es} = \frac{\Delta H_{m,CPCM} + \Delta H_{c,CPCM}}{\Delta H_{m,PCM} + \Delta H_{c,PCM}} \times 100\%, \tag{S5}$$

$$C_{es} = \frac{\left(\Delta H_{m,CPCM} + \Delta H_{c,CPCM}\right)\Delta H_{m,PCM}}{\left(\Delta H_{m,PCM} + \Delta H_{c,PCM}\right)\Delta H_{m,CPCM}} \times 100\%, \tag{S6}$$

where $\Delta H_{m,CPCM}$ and $\Delta H_{c,CPCM}$ represent the melting and crystallization enthalpies of CPCM, respectively, while $\Delta H_{m,PCM}$ and $\Delta H_{c,PCM}$ represent the melting and crystallization enthalpies of PCM, respectively. The PCM leakage was characterized by placing CPCM (4 cm×4 cm×0.2 cm) onto an oil absorbing paper under a 100 g weight (2.6 kPa). The leakage percentage was calculated by the PCM mass ratio before and after one melting-solidification cycle.

Photothermal tests

A solar simulator (SciSun-300, Sciencetech) equipped with an air mass AM1.5G filter and a xenon light source was used to illuminate the sample with a light intensity of 1 kW/m^2 (1.0 sun illumination). A T-type thermocouple was attached to the back of the sample (4 cm × 4 cm) and connected to a data acquisition device (KAIPUSEN 640L) for detecting the bottom temperature variation. A handheld infrared camera (FLIR E75, America) was used to take infrared photos and record surface temperatures. The photothermal experiment was conducted at both room temperature (22 °C) and a cold environment (–20 °C).

Supplementary Figures

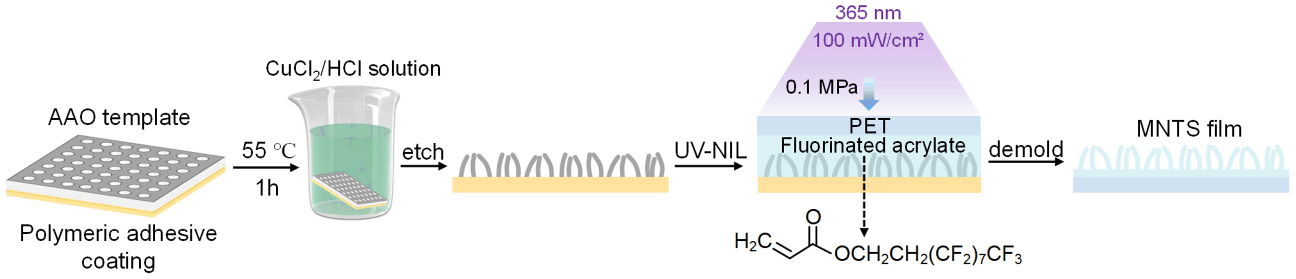

Supplementary Fig. 1. Fabrication procedures of moth-eye nanostructured transparent superhydrophobic (MNTS) film.

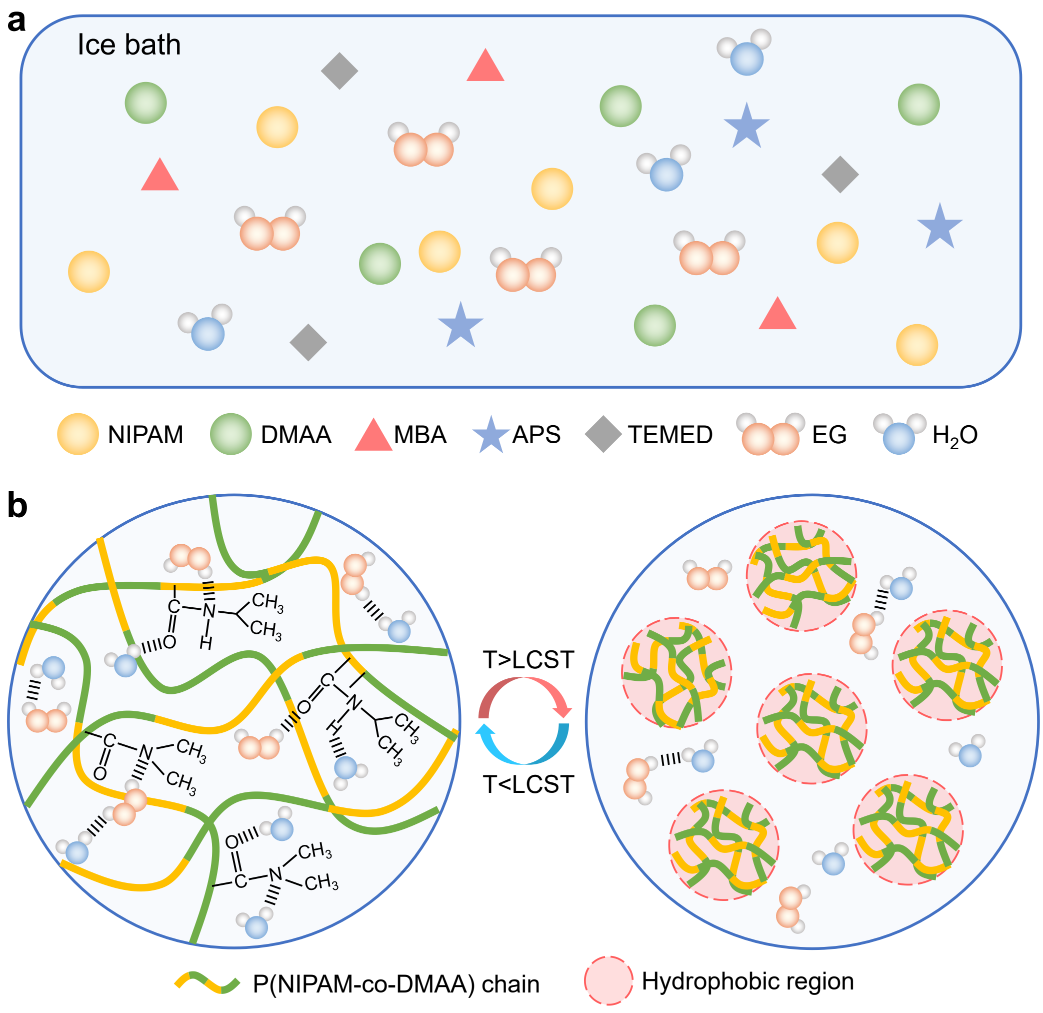

Supplementary Fig. 2. **a** Preparation mechanism and **b** phase transition mechanism diagram of PNDE hydrogels.

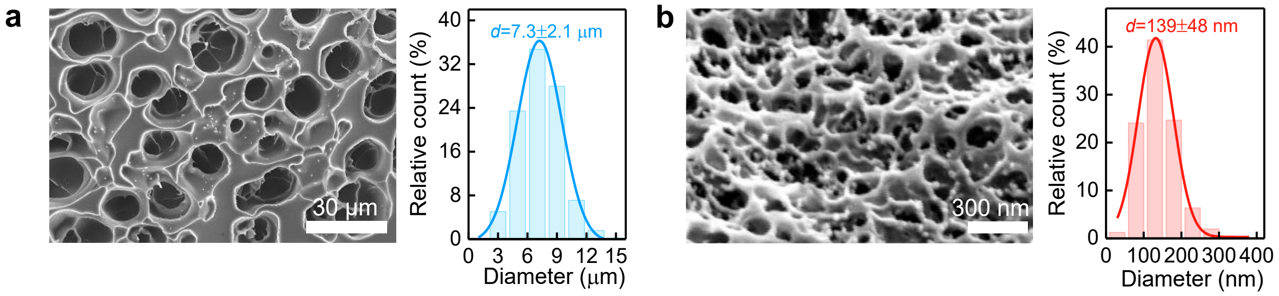

Supplementary Fig. 3. Scanning electron microscopy (SEM) images and pore diameter distribution of freeze-dried PNIPAM hydrogels **a** before and **b** after phase transition. The SEM measurements were independently repeated three times with consistent results.

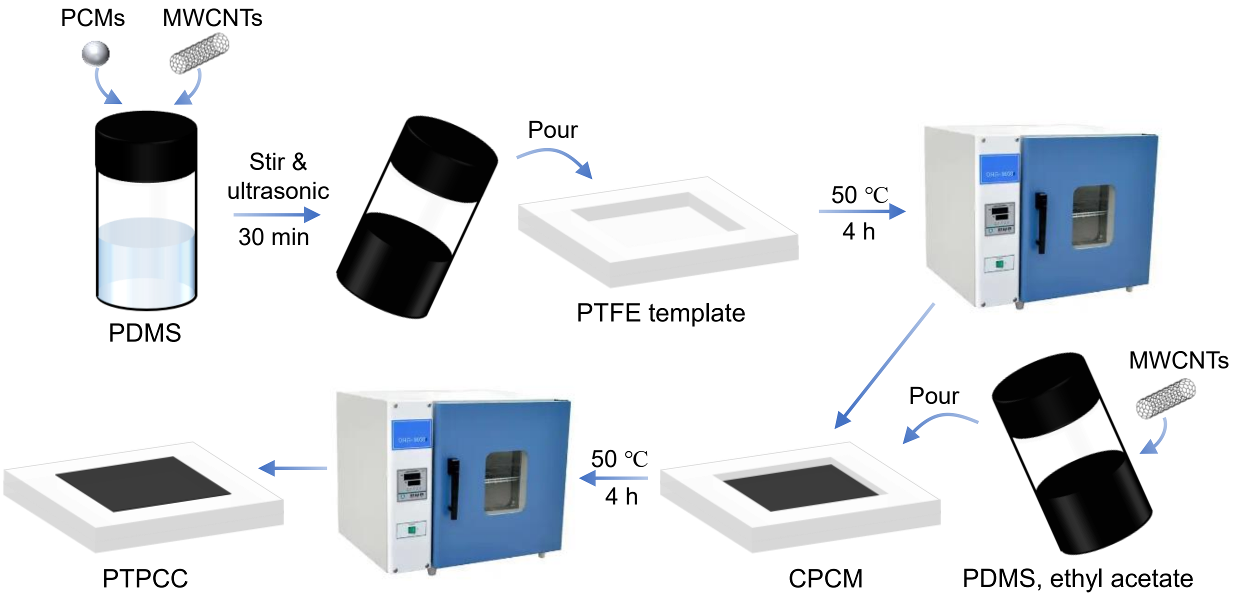

Supplementary Fig. 4. Fabrication procedures of photothermal phase change composite (PTPCC).

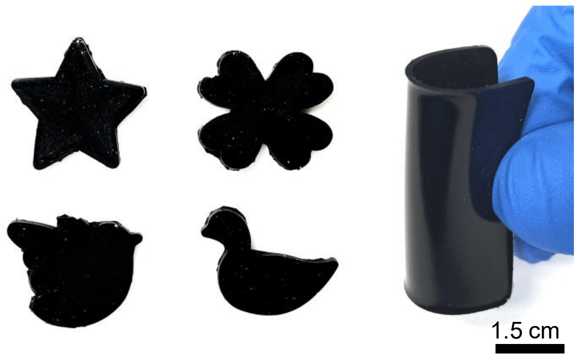

Supplementary Fig. 5. Photographs of patterned and bent phase change composite (PTPCC).

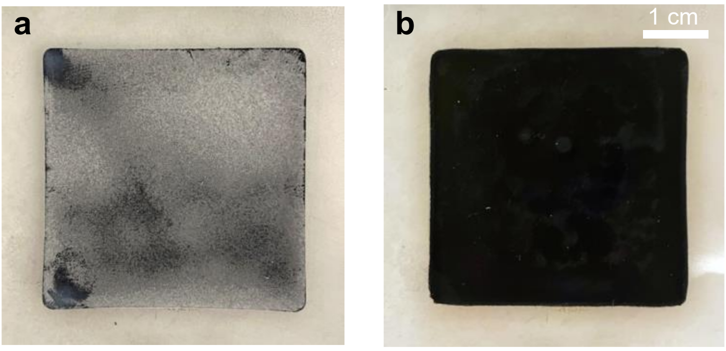

Supplementary Fig. 6. Photographs of **a** composite phase-change material (CPCM) and **b** photothermal phase change composite (PTPCC) after placing in a cold chamber at –20 °C.

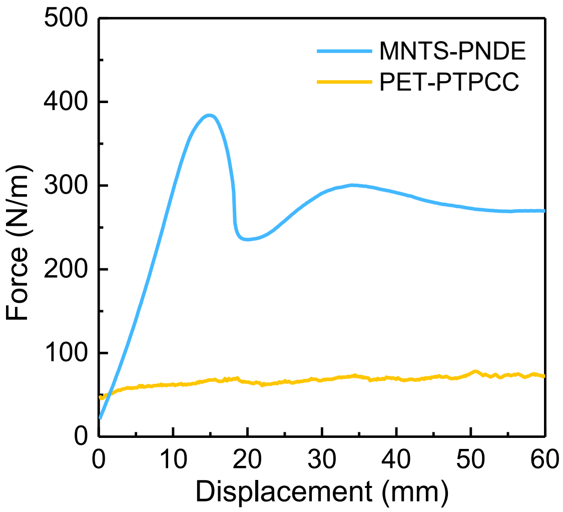

Supplementary Fig. 7. Peel force versus displacement for moth-eye nanostructured transparent superhydrophobic (MNTS) film-PNDE hydrogel and polyethylene terephthalate (PET) film-photothermal phase change composite (PTPCC) interfaces

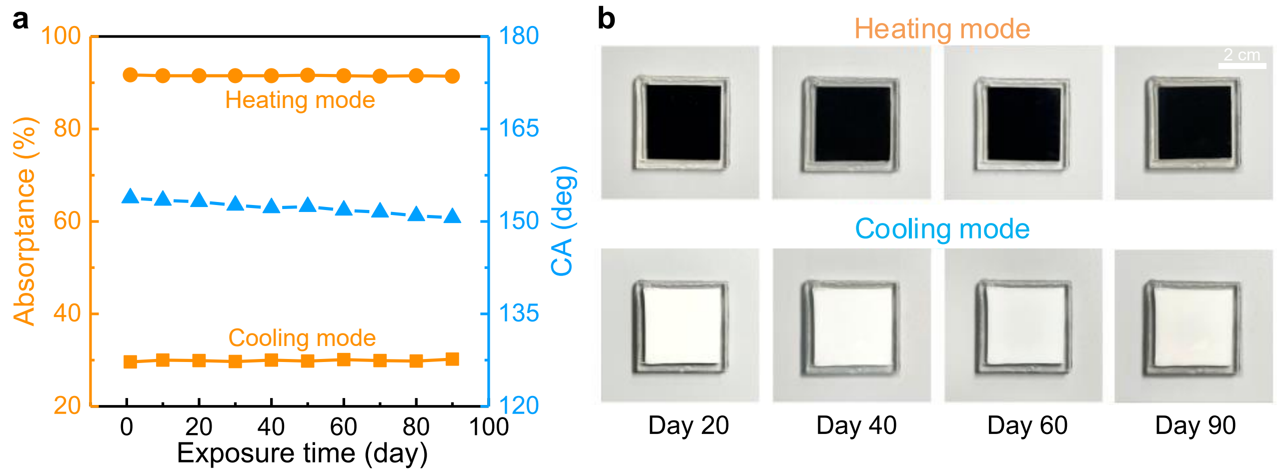

Supplementary Fig. 8. Changes in **a** solar absorption, contact angle (CA) and **b** optical images of the temperature-adaptive photothermal storage superhydrophobic (TAPSS) film under outdoor durability tests.

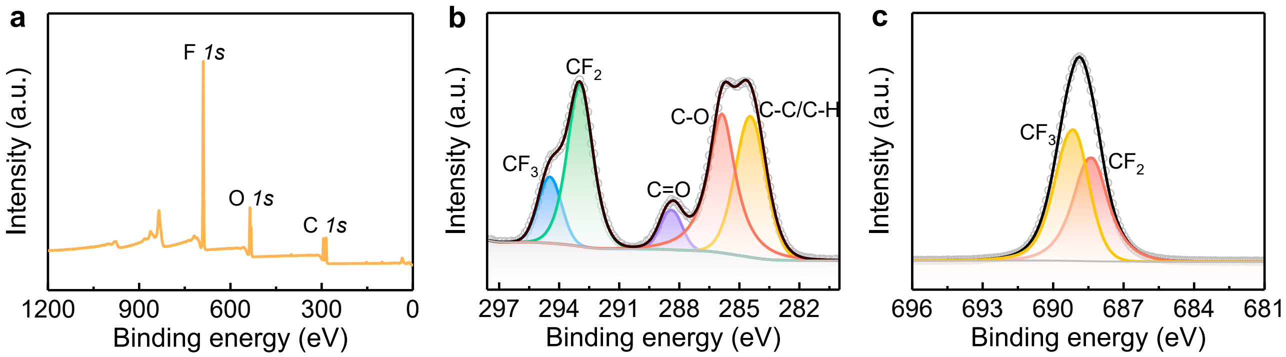

Supplementary Fig. 9. **a** X-ray photoelectron spectroscopy (XPS) survey spectrum and high-resolution spectra of **b** C 1s and **c** F 1s for moth-eye nanostructured transparent superhydrophobic (MNTS) film. The results indicate characteristic peaks of C *1s* (285.1 eV), O *1s* (532.5 eV) and F *1s* (689.0 eV).

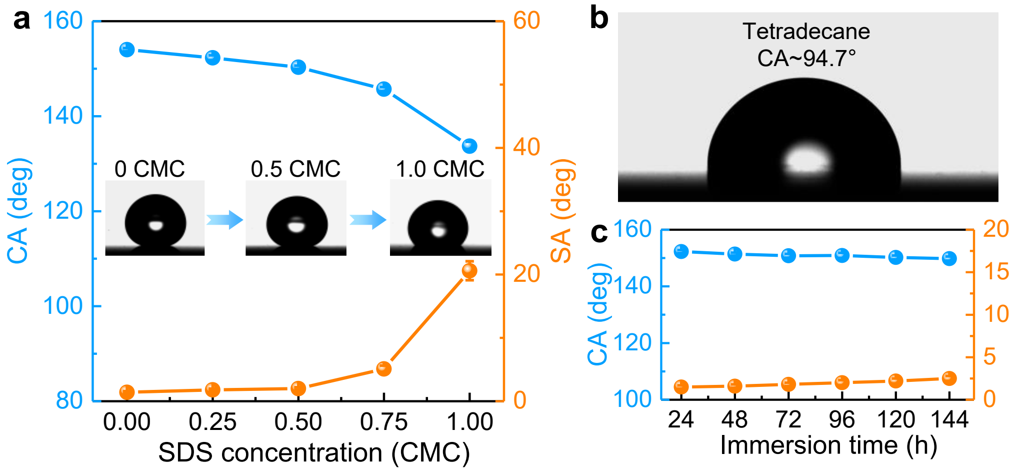

Supplementary Fig. 10. a Changes in contact angle (CA) and sliding angle (SA) of sodium dodecyl sulfate (SDS) aqueous solution droplets with different concentration. **b** CA of a tetradecane droplet on the MNTS film. **c** Changes in CA and SA of water droplets after immersion in tetradecane. Dot heights, mean values; error bards, standard deviation (*n*=5).

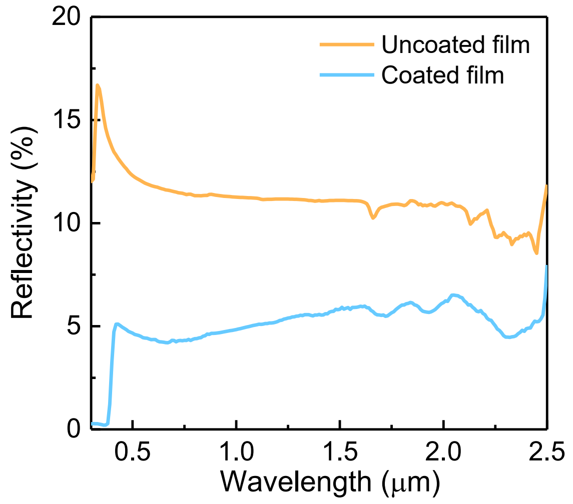

Supplementary Fig. 11. Reflectivity spectra of moth-eye nanostructured transparent superhydrophobic (MNTS)-coated and uncoated polyethylene terephthalate (PET) films.

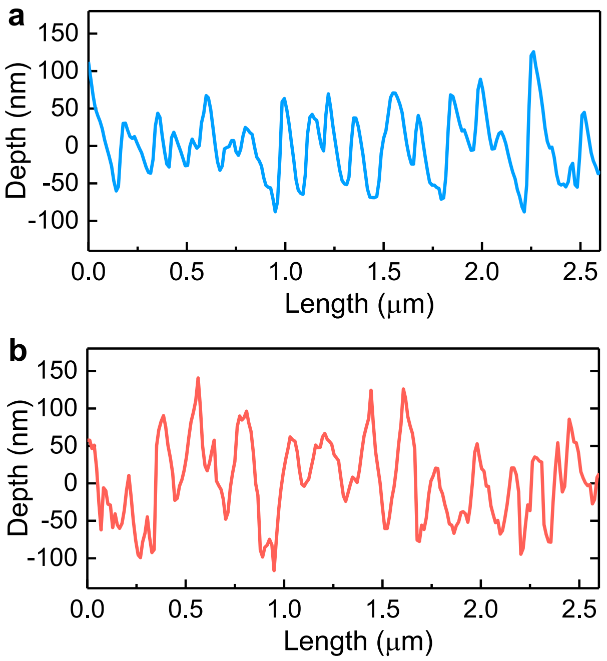

Supplementary Fig. 12. Line profiles of moth-eye nanostructured transparent superhydrophobic (MNTS) film extracted from atomic force microscopy (AFM) tests. The lines in **a** and **b** are perpendicular to each other.

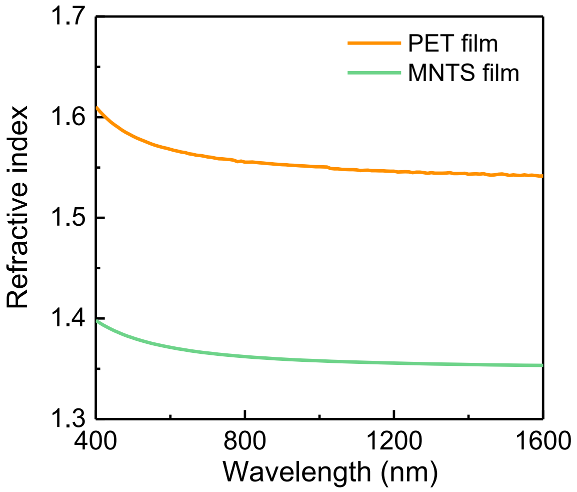

Supplementary Fig. 13. Refractive index of polyethylene terephthalate (PET) film and moth-eye nanostructured transparent superhydrophobic (MNTS) film.

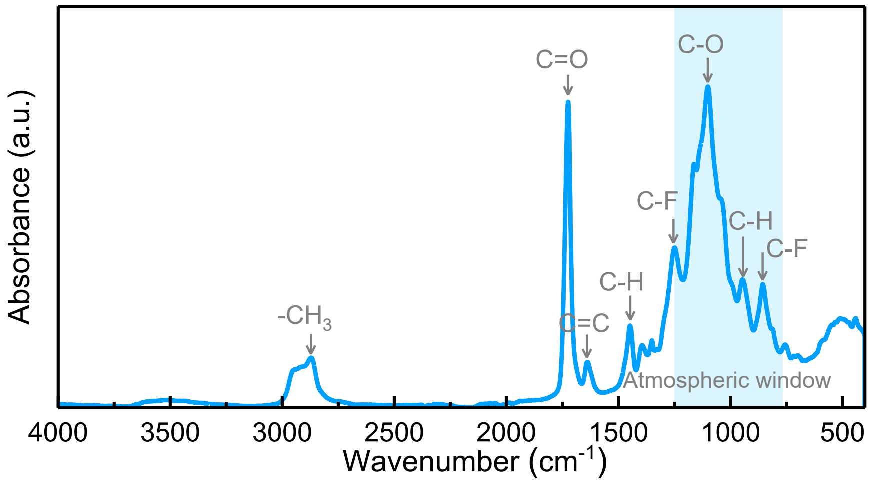

Supplementary Fig. 14. Attenuated total reflectance-Fourier transform infrared (ATR-FTIR) spectrum of moth-eye nanostructured transparent superhydrophobic (MNTS) film.

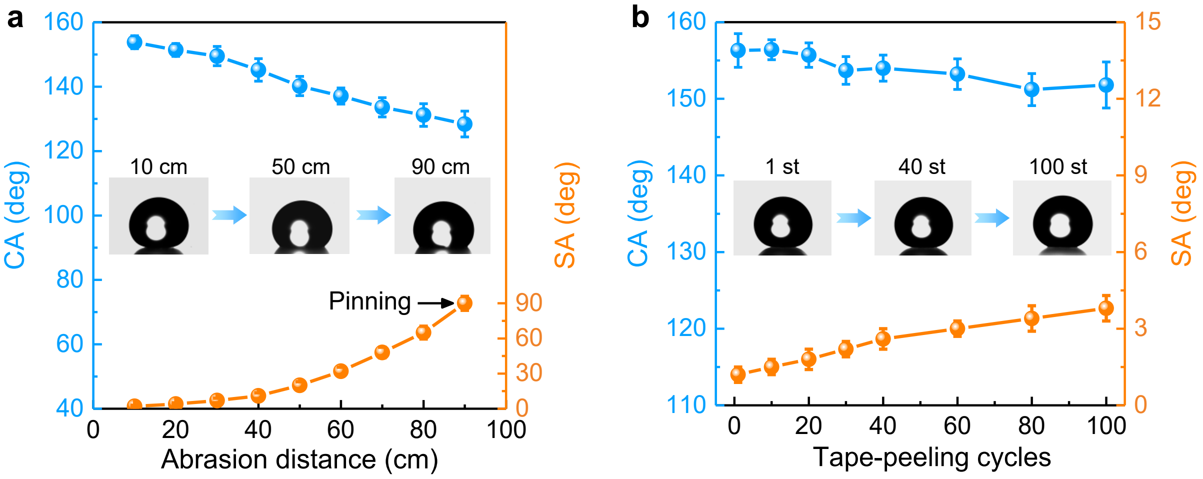

Supplementary Fig. 15. Changes in contact angle (CA) and sliding angle (SA) of Glaco coating during **a** sandpaper abrasion and **b** tape-peeling tests. Dot heights, mean values; error bards, standard deviation (n=5).

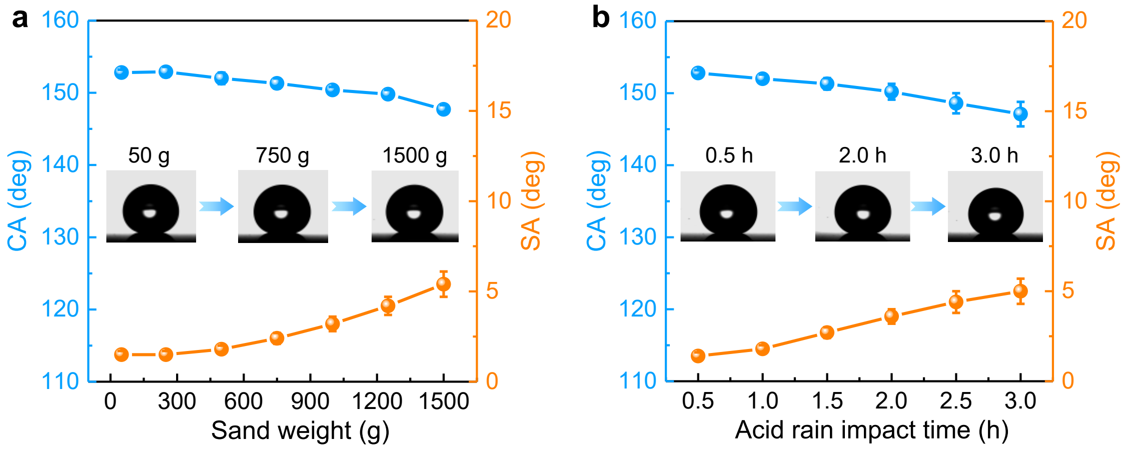

Supplementary Fig. 16. Changes in contact angel (CA) and sliding angle (SA) of moth-eye nanostructured transparent superhydrophobic (MNTS) film during **a** sand impact and **b** acid rain impact tests. Dot heights, mean values; error bards, standard deviation (n=5).

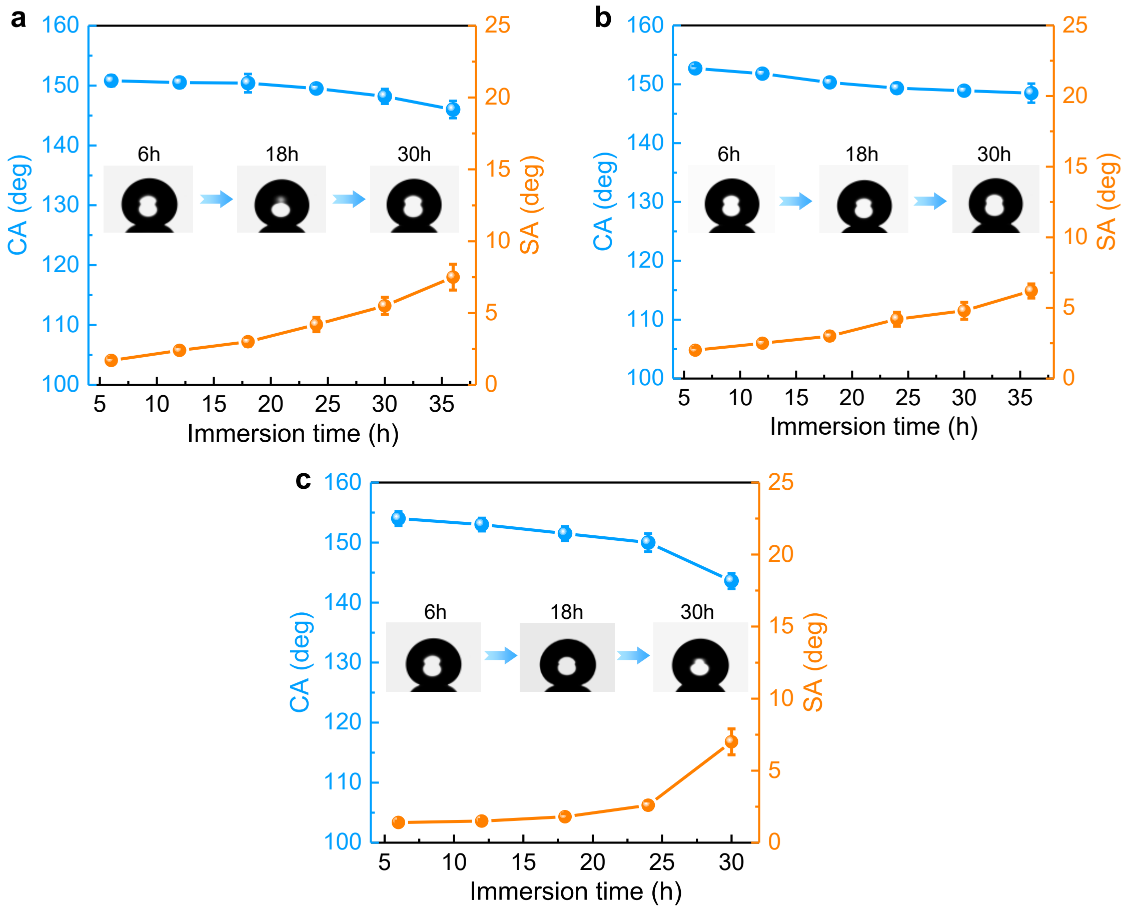

Supplementary Fig. 17. Chemical stability of moth-eye nanostructured transparent superhydrophobic (MNTS) film. Changes in contact angel (CA) and sliding angle (SA) after immersion in **a** HCl solution (pH=2), **b** NaOH solution (pH=12) and **c** 1 M NaCl solution. Dot heights, mean values; error bards, standard deviation (n=5).

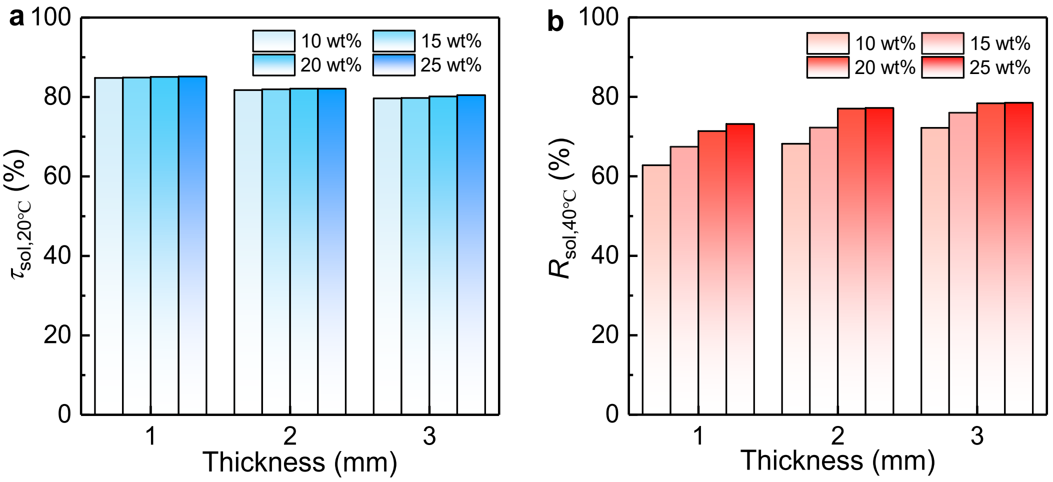

Supplementary Fig. 18. a Solar transmittance at 20 °C ($\tau_{sol,20°C}$) and **b** solar reflectivity at 40 °C ($R_{sol,40°C}$) of PNIPAM hydrogels as a function of thickness and NIPAM concentration.

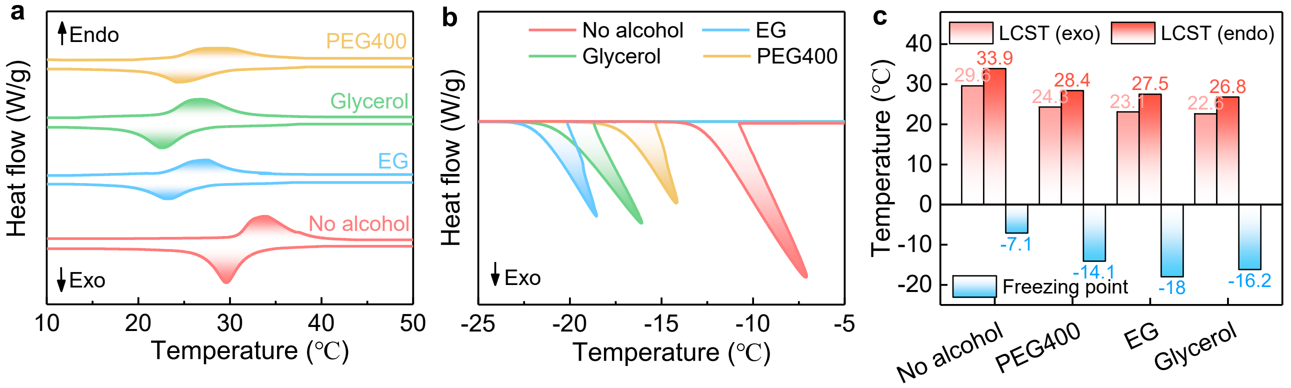

Supplementary Fig. 19. Phase transition and anti-freezing performance of pure PNIPAM hydrogel and those adding 15 wt% PEG400, ethanol glycol, glycerol. DSC diagrams for **a** lower critical solution temperature (LCST) and **b** freezing point. **c** Summary for LCST and freezing point values.

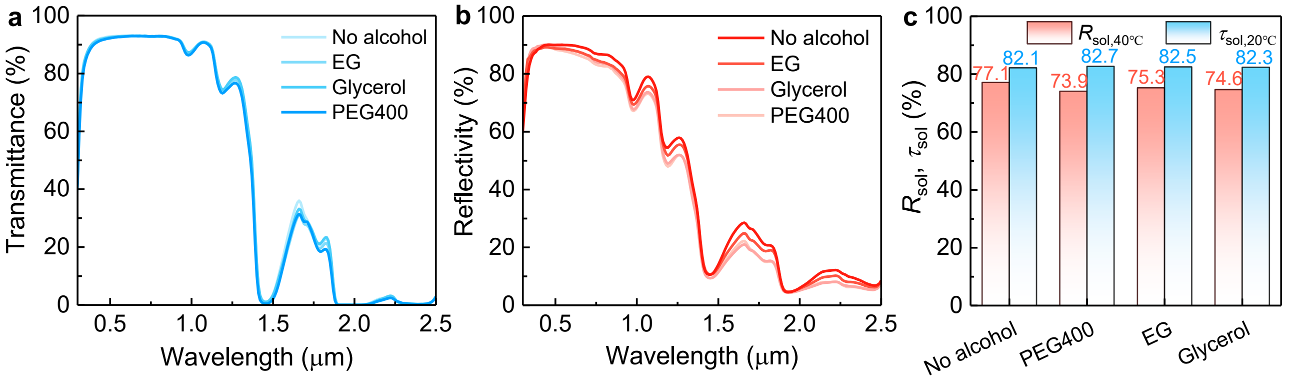

Supplementary Fig. 20. Optical performance of pure PNIPAM hydrogel and those adding 15 wt% PEG400, ethanol glycol, glycerol. **a** Solar transmittance spectra at 20 °C and **b** solar reflectivity spectra at 40 °C. **c** Summary of the values for $R_{sol,40°C}$ and $\tau_{sol,20°C}$.

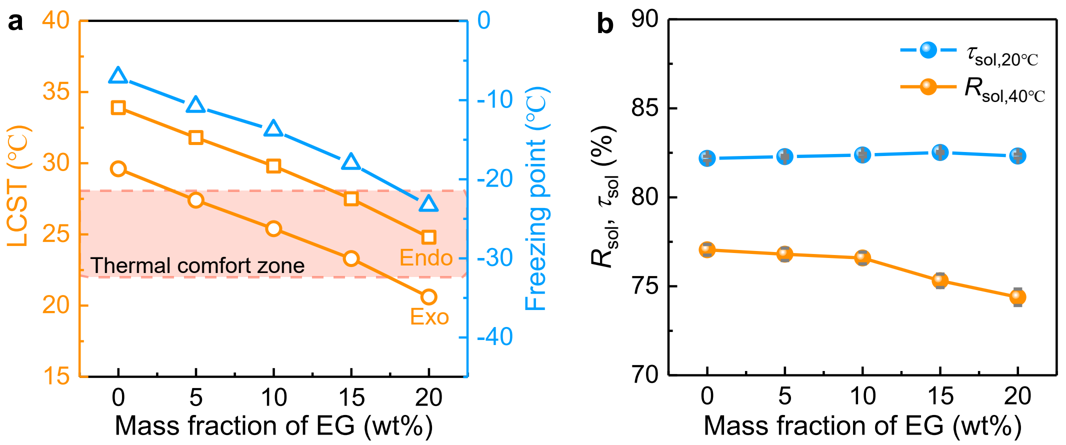

Supplementary Fig. 21. **a** Lower critical solution temperature (LCST), freezing point, **b** $\tau_{sol,20°C}$ and $R_{sol,40°C}$ as a function of ethylene glycol mass fraction. Dot heights, mean values; error bards, standard deviation (n=3).

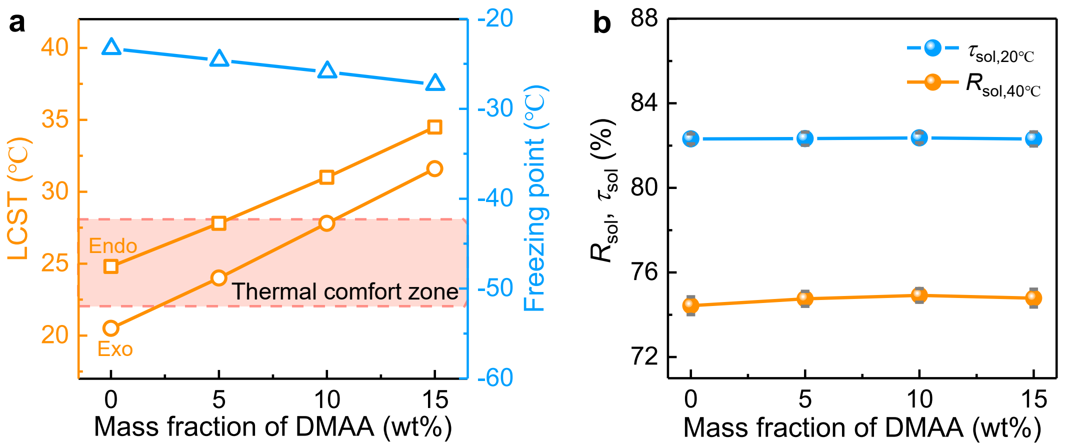

Supplementary Fig. 22. **a** Lower critical solution temperature (LCST), freezing point, **b** $\tau_{sol,20°C}$ and $R_{sol,40°C}$ as a function of DMAA mass fraction. The mass fraction of EG is fixed at 20 wt%. Dot heights, mean values; error bards, standard deviation (n=3).

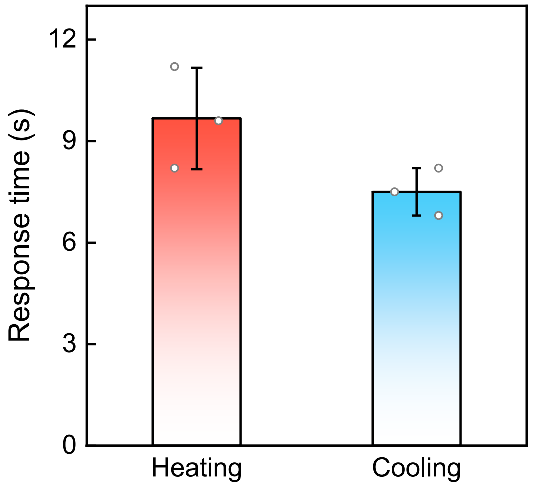

Supplementary Fig. 23. Response time of PND_5E_{20} hydrogels during cooling process (20 °C) and heating process (40 °C). Bar heights, mean values; error bards, standard deviation (*n*=3).

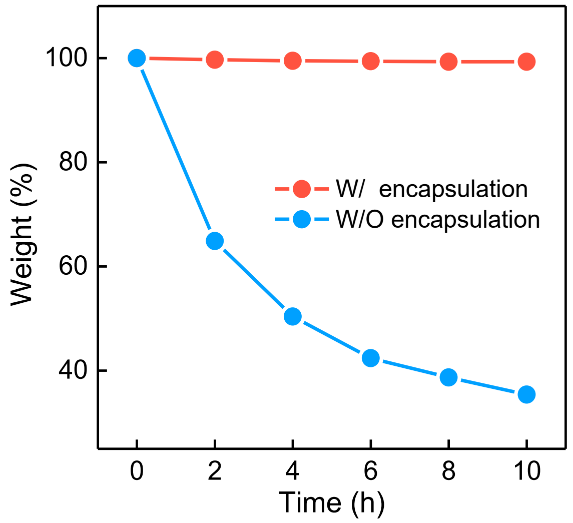

Supplementary Fig. 24. Weight change of PND_5E_{20} hydrogels during heating process (40 °C) for 10 hours.

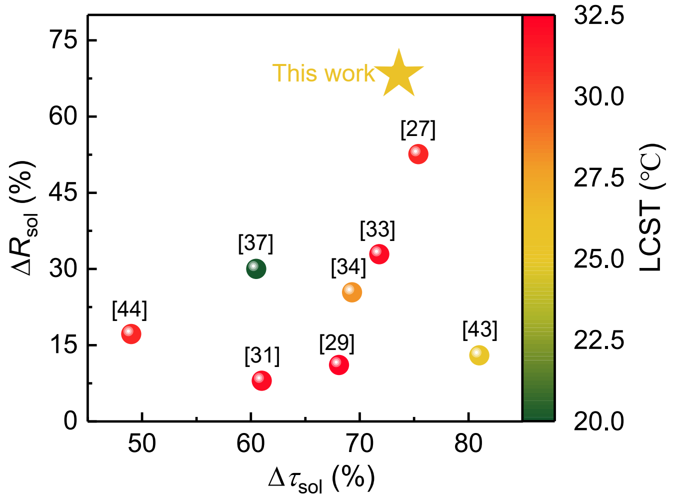

Supplementary Fig. 25. Comparison of transmittance modulation ($\Delta\tau_{sol}$), reflectivity modulation (ΔR_{sol}) and LCST values for thermochromic hydrogels.

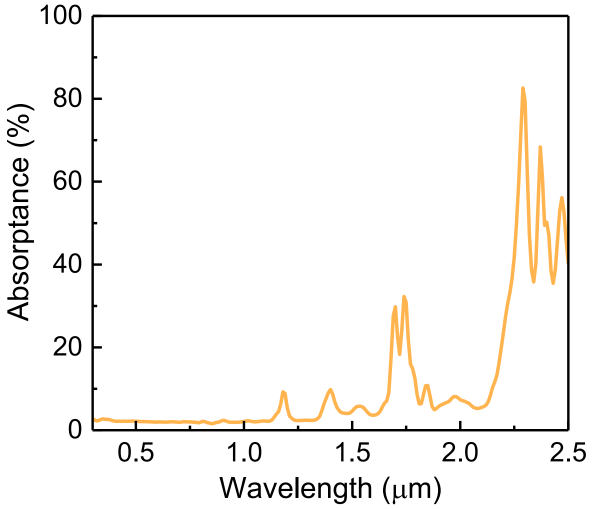

Supplementary Fig. 26. Solar absorptance spectrum of polydimethylsiloxane (PDMS) film.

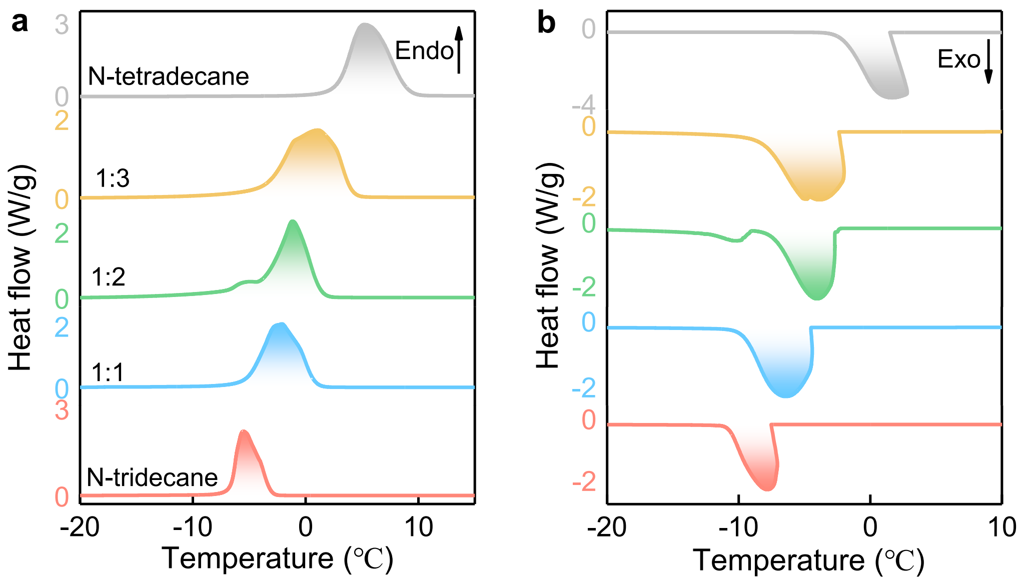

Supplementary Fig. 27. Differential scanning calorimeter thermograms of phase change materials with different mass ratio of n-tridecane to n-tetradecane during **a** heating and **b** cooling processes.

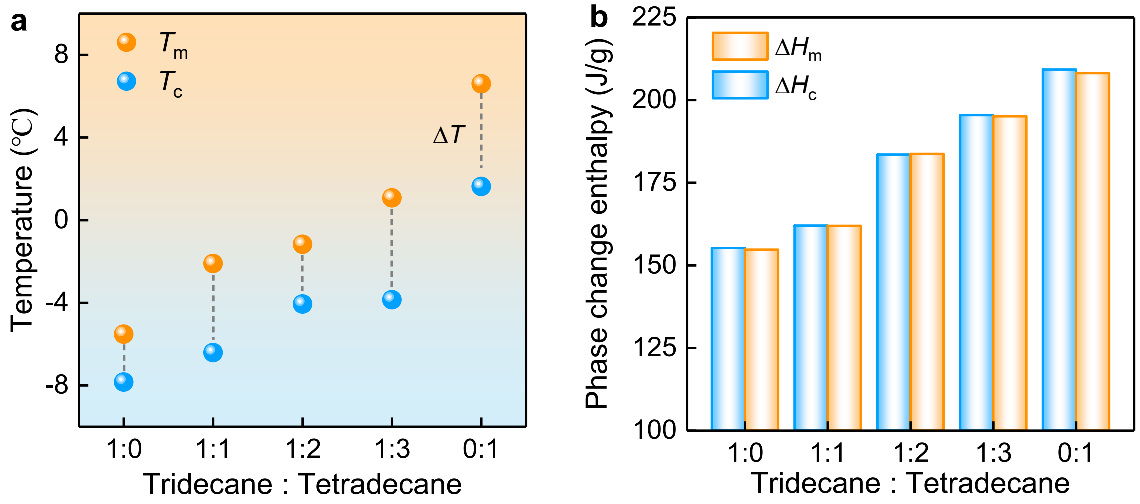

Supplementary Fig. 28. a Phase change temperature and **b** phase change enthalpy of phase change materials as a function of the mass ratio of n-tridecane to n-tetradecane.

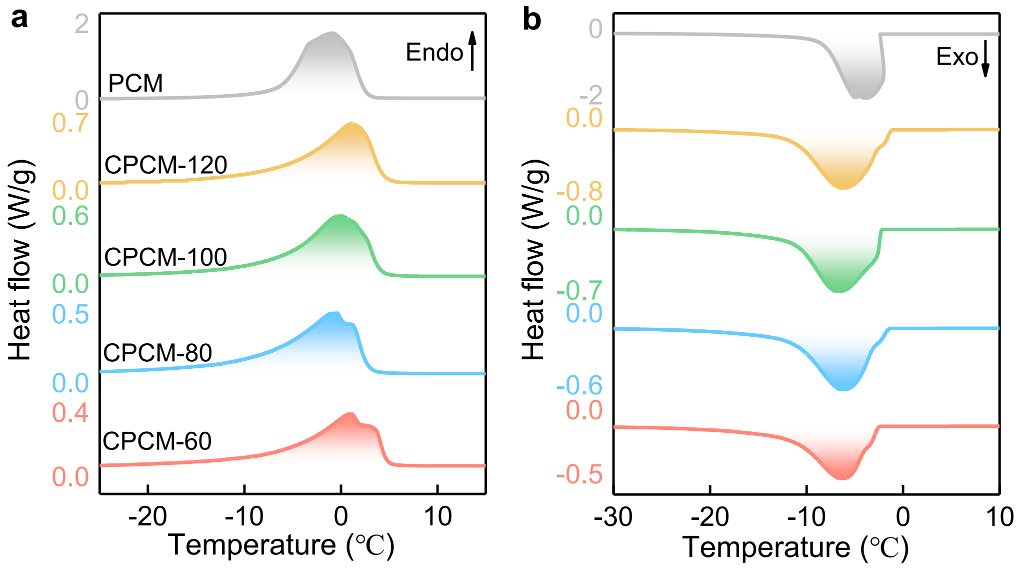

Supplementary Fig. 29. Differential scanning calorimeter thermograms of composite phase-change materials (CPCMs) with different mass ratio of phase change material to polydimethylsiloxane (PDMS) during **a** heating and **b** cooling processes.

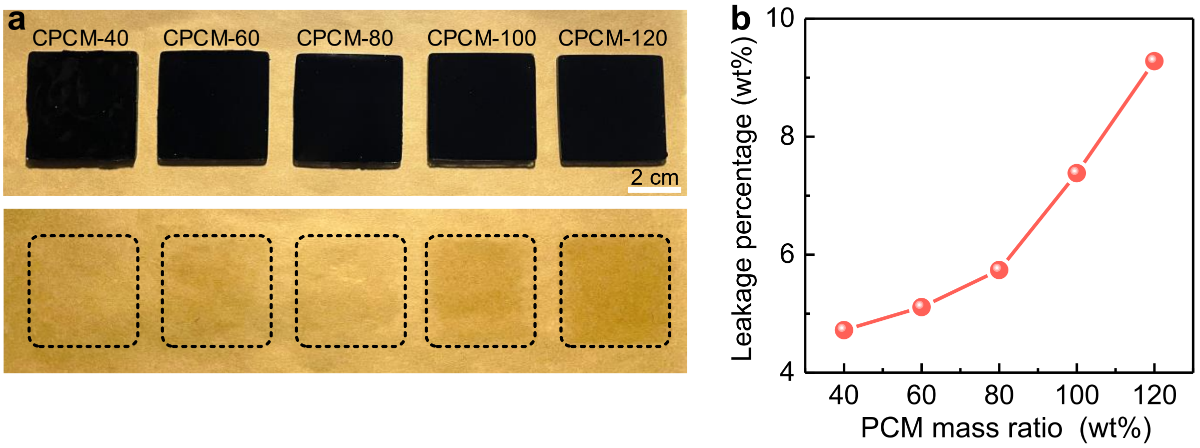

Supplementary Fig. 30. **a** Photographs and **b** leakage percentage for composite phase-change materials (CPCMs) with different mass ratio (m_{PCM}:m_{PDMS}) after one solidification-melting cycle under 2.6 kPa.

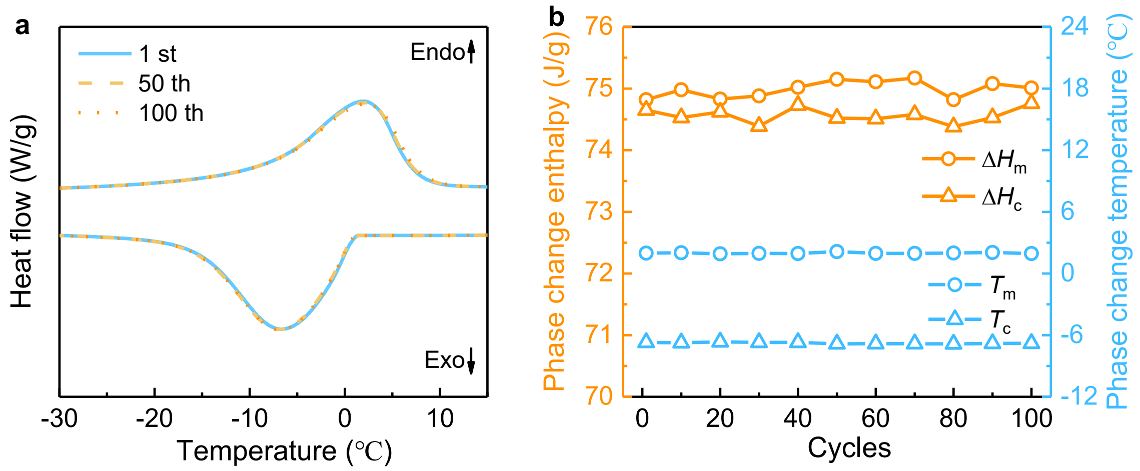

Supplementary Fig. 31. a DSC curves, **b** phase change enthalpies and temperatures of composite phase-change materials loaded with 80 wt% PCM during 100 melting-solidification cycles.

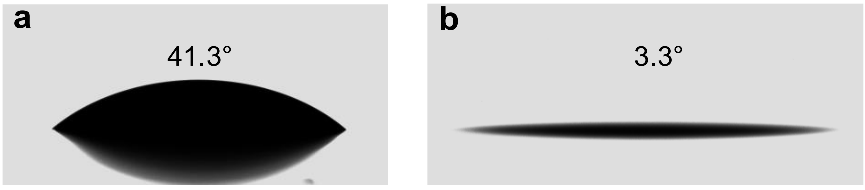

Supplementary Fig. 32. Contact angle of phase change material (PCM) droplets on **a** PT-6 film and **b** aluminum plate.

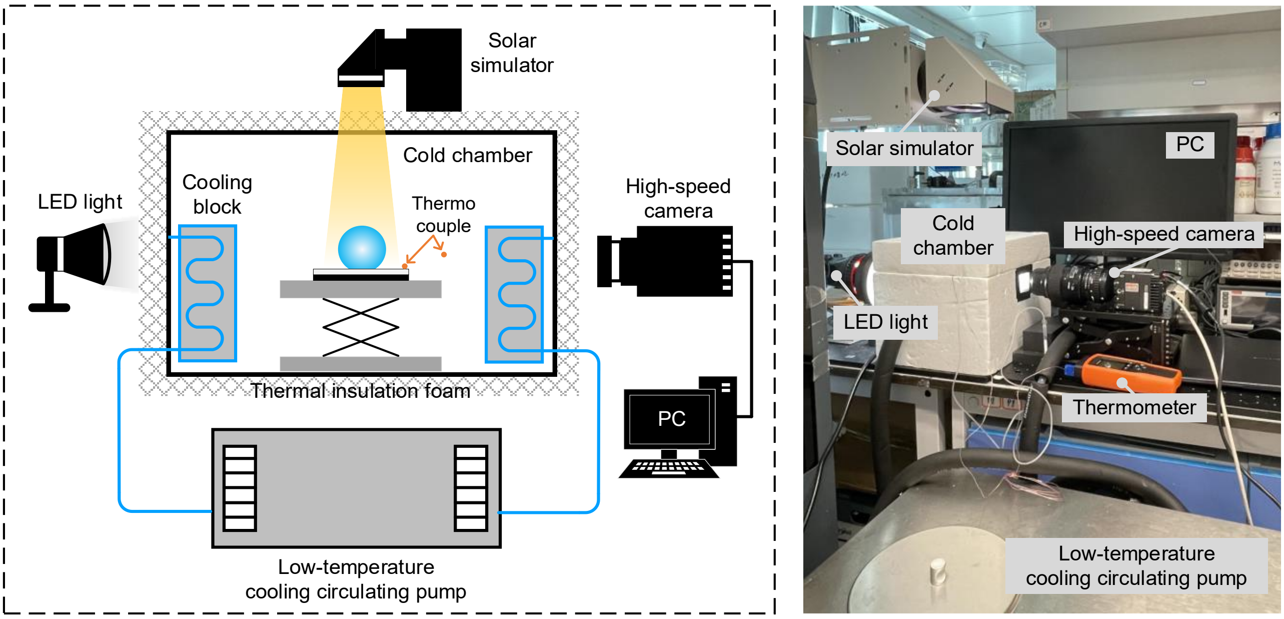

Supplementary Fig. 33. Schematic diagram and physical photograph of the experimental setup designed for anti-icing and photothermal deicing tests.

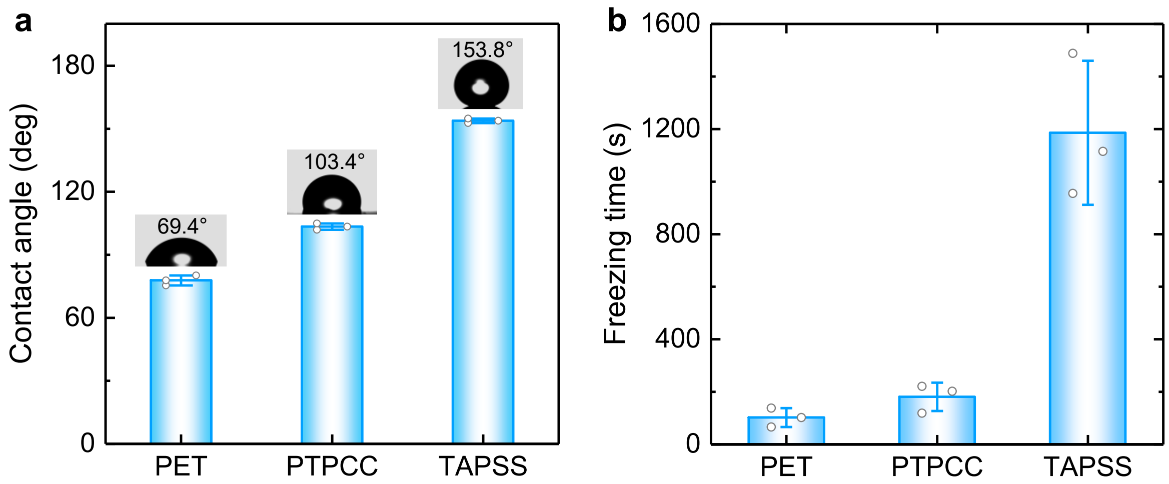

Supplementary Fig. 34. a Water contact angle at room temperature and **b** freezing time (–20°C, 20% RH) on polyethylene terephthalate (PET), photothermal phase change composite (PTPCC) and temperature-adaptive photothermal storage superhydrophobic (TAPSS) films. Bar heights, mean values; error bards, standard deviation (*n*=3).

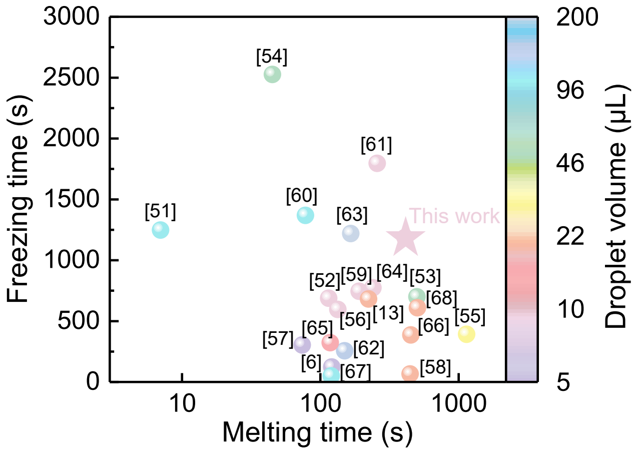

Supplementary Fig. 35. Comparison of freezing time and melting time for a water droplet on photothermal superhydrophobic surfaces (–20 °C, 1.0 sun illumination).

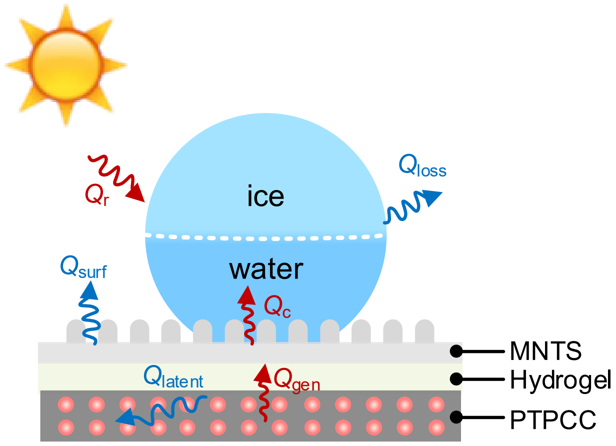

Supplementary Fig. 36. Schematic diagram of heat transfer mechanism during ice melting on temperature-adaptive photothermal storage superhydrophobic (TAPSS) film.

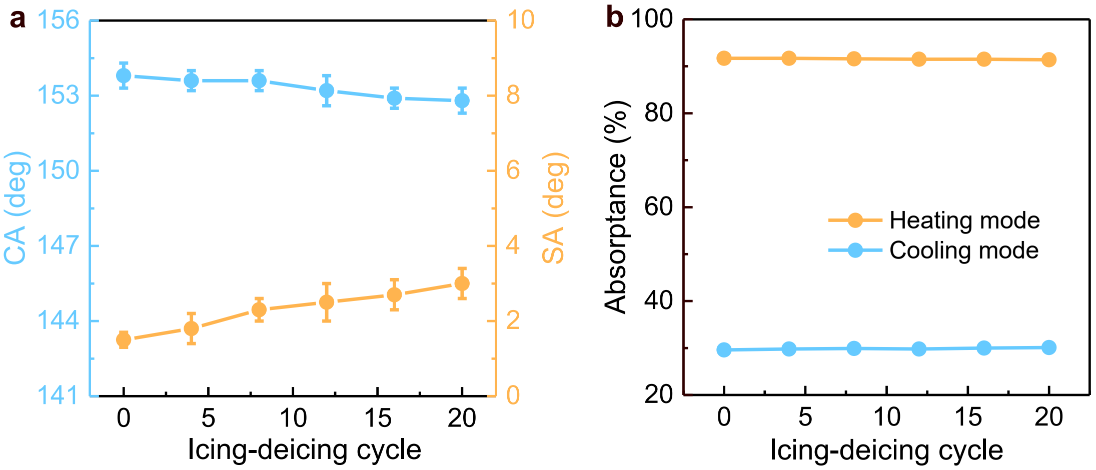

Supplementary Fig. 37. Variation of **a** surface wettability and **b** optical performance for temperature-adaptive photothermal storage superhydrophobic (TAPSS) film after multiple icing-deicing cycles. Dot heights, mean values; error bards, standard deviation (*n*=5).

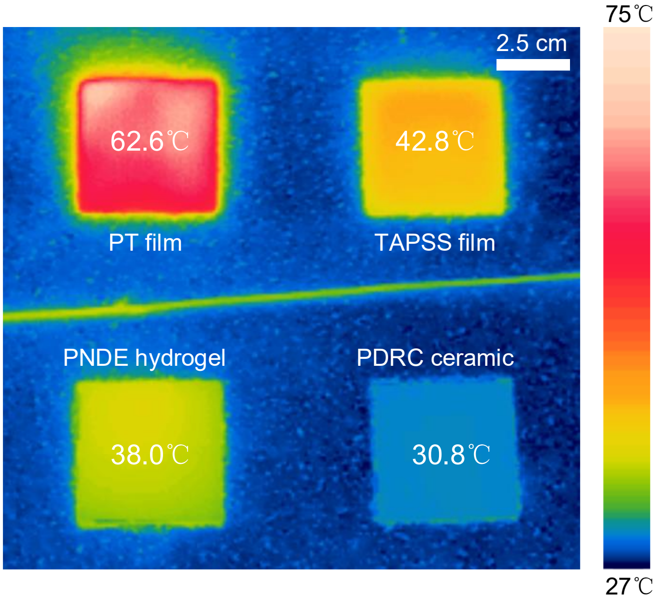

Supplementary Fig. 38. Infrared images of samples measured at 10:30 on 8 June 2025 in Hong Kong.

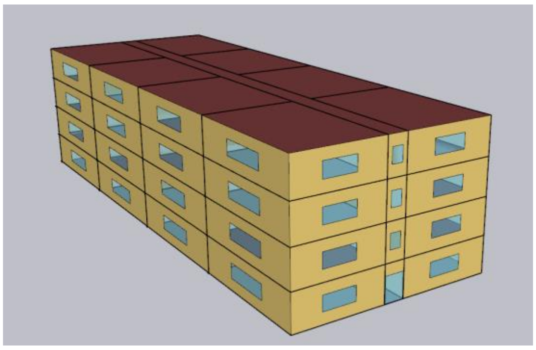

Supplementary Fig. 39. Mid-rise apartment model used in the EnergyPlus simulation.

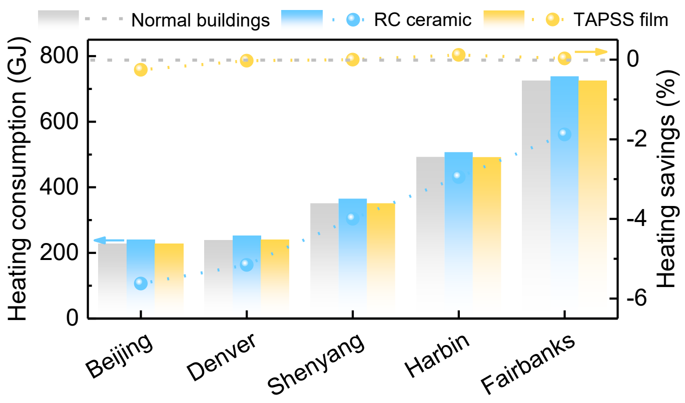

Supplementary Fig. 40. Annual heating energy consumption and savings for buildings equipped with temperature-adaptive photothermal storage superhydrophobic (TAPSS) and radiative cooling (RC) ceramic roofs, and normal buildings across cities in various climate zones.

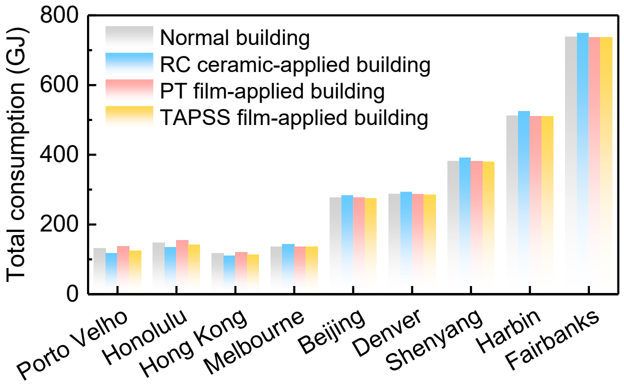

Supplementary Fig. 41. Annual total energy consumption of temperature-adaptive photothermal storage superhydrophobic (TAPSS) film-applied buildings, photothermal (PT) film-applied buildings, radiative cooling (RC) ceramic-applied buildings, and normal buildings across cities in various climate zones.

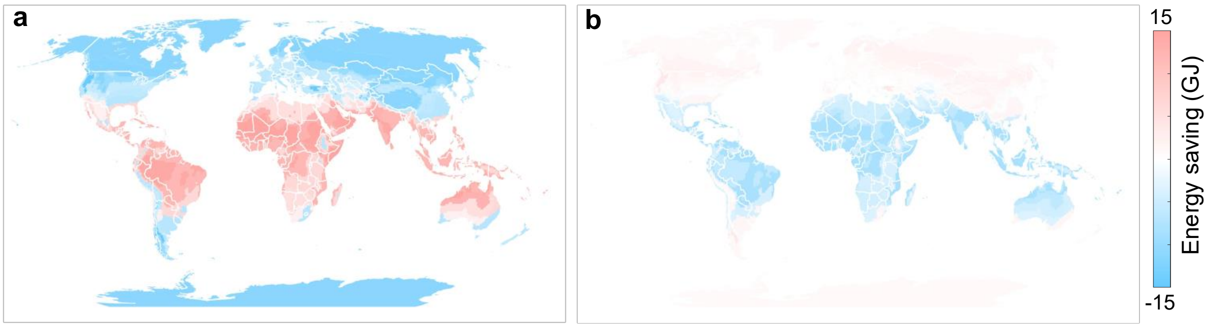

Supplementary Fig. 42. Global energy-saving potential maps showing net energy consumption differences between **a** radiative cooling ceramic-equipped, **b** photothermal film-equipped and conventional buildings.

Supplementary Tables

Supplementary Table 1. Performance of representative functional materials for photothermal anti-/deicing in the literature.

Materials	CA (deg)	$\alpha_{sol,heat}$ (%)	$\Delta\alpha_{sol}$ (%)	ΔH (J/g)	$T_{m/c}$ (°C)	Ref.
Photothermal superhydrophobic materials (PSMs)	165	97	/	/	/	[1]
	157	93	/	/	/	[2]
	166	97	/	/	/	[3]
	163	99	/	/	/	[4]
	162	95	/	/	/	[5]
	163	93	/	/	/	[6]
Photothermal storage superhydrophobic materials (PSSMs)	158	NA	/	68	35	[7]
	NA	93	/	100	2.6	[8]
	NA	95	/	42	-2.2	[9]
	156	97	/	111	4.5	[10]
	153	NA	/	33	-5	[11]
	166	NA	/	35	3.5	[12]
Thermochromic microcapsule-based materials (TCMMs)	114	84	32	NA	NA	[13]
	NA	NA	48	83	22	[14]
	104	43	33	NA	26.5	[15]
	124	69	31	NA	28	[16]
	110	38	30	NA	22	[17]
	163	21	13	NA	28	[18]
TAPSS film	154	92	62	75	-2.4	This work

Supplementary Table 2. Performance of representative transparent superhydrophobic surfaces in the literature

Superhydrophobicity	Optical performance	Mechanical durability	Chemical stability	UV resistance	Ref.
CA=168° SA=2°	79.5-92.4% in the visible spectra	20 tape-peeling cycles	1 M NaCl, NaOH, and HCl aqueous solution for 72 h	/	[19]
CA=161° SA=1°	85% in the visible spectra	300 cm sandpaper abrasion	pH=1 and 13 for 72 h	40 h	[20]
CA=164° SA<1°	91.4% in the visible spectra	900 cm sandpaper abrasion	pH=1 and 13 for 72 h	720 h	[21]
CA=162° SA=1.5°	89.3% in the visible spectra	800 cm sandpaper abrasion and 200 tape-peeling cycles	pH=0 and 14 for 72 h	72 h	[22]
CA=153° SA=5.1°	98% in the visible spectra	20 tape-peeling cycles	pH=1 for 3 h	60 h	[23]
CA=154° SA=3.2°	92.5% in the visible spectra	15 cm sandpaper abrasion	pH=0 for 3 h	/	[24]
CA=159° SA=1°	87% in the visible spectra	/	pH=1 and 14 for 432 h	1440 h	[25]
CA=154° SA=1.5°	τ_{sol}=90.9%, τ>93% in the visible spectra	200 cm sandpaper abrasion and 100 tape-peeling cycles	1 M NaCl, pH=2 and 12 for 24 h	672 h	This work

Supplementary Table 3. Performance of representative thermochromic hydrogels in the literature.

Hydrogels	Solvents	$\Delta\tau_{sol}$ (%)	ΔR_{sol} (%)	LCST (°C)	Freezing point (°C)	Response time (min)	Ref.
PNIPAM	Water	69.1	NA	32	NA	NA	[26]
PNIPAM	Water	75.4	52.6	31	NA	NA	[27]
PNIPAM	Water	68.3	NA	31	NA	NA	[28]
PNIPAM	Water	68.1	11.1	32.5	NA	15	[29]
PNIPAM	Water	55.2	NA	34.0	NA	3	[30]
PNIPAM	Water	61	8	32	NA	NA	[31]
PNIPAM	Water/glycerol	60.8	NA	26	-18	0.16	[32]
PNIPAM	Water/ethanol	71.8	32.9	28	NA	0.5	[33]
PNIPAM	Water/ethanol	69.3	25.4	28	NA	0.23	[34]
P(NIPAM-co-AEMA)	Water	81.3	NA	32	NA	5	[35]
P(NIPAM-co-AAm)	Water/glycerol	62.1	NA	24	-15	NA	[36]
P(NIPAM-co-AAm)	Water	60.5	30	20	NA	NA	[37]
P(NIPAM-co-AAm)	Water	38.1	NA	22.9	NA	2.0	[38]
P(NIPAM-co-AAm-AA)	Water	69.5	NA	31	NA	0.33	[39]
P(NIPAM-co-ACMO)	Water	79.3	NA	30	NA	NA	[40]
P(NIPAM-co-DMAA)	Water	88.8	NA	40.7	NA	NA	[41]
P(NIPAM-co-DMAA)	Water/EG	80.7	NA	30	-27	NA	[42]
P(NIPAM-co-DMAA)	Water/glycerol	81	13	24-43	NA	6	[43]
HPC	Water	49.0	17.2	31	NA	NA	[44]
HPC	NaCl solution	43.6	NA	31	NA	NA	[45]
HPC-PAA	Water	47.5	NA	26.5	NA	1.4	[46]
HPC-PAA-PAAm	Water	54.0	NA	30	NA	NA	[47]
PNIPAM-HPC	Water	57.2	NA	28.5	NA	0.1	[48]
PNIPAM-HPC	Water	64.5	NA	29	-13	0.28	[49]
PVB	LiCl solution	80.8	NA	28.5	-15	0.16	[50]
P(NIPAM-co-DMAA)	Water/EG	73.6	68.4	25.9	-24.6	0.15	This work

Supplementary Table 4. Phase change and encapsulation properties of composite phase-change materials (CPCMs) with different mass ratio of phase change materials to polydimethylsiloxane (PDMS).

Samples	T_c (°C)	T_m (°C)	ΔT (°C)	ΔH_c (J/g)	ΔH_m (J/g)	E_{en}	E_{es}	C_{es}
PCM	-3.85	1.08	4.93	195.45	195.08	/	/	/
CPCM-120	-6.31	2.85	9.16	93.33	93.22	47.79	47.77	99.96
CPCM-100	-6.85	1.87	8.72	87.13	87.02	44.61	44.59	99.97
CPCM-80	-6.34	1.5	7.84	75.09	75.01	38.45	38.43	99.96
CPCM-60	-6.46	1.05	7.51	60.87	60.89	31.21	31.18	99.89

Supplementary Table 5. Anti-/deicing performance of representative photothermal superhydrophobic surfaces in the literature (-20 °C, 1.0 sun illumination).

Materials	Droplet volume (μL)	Freezing time (s)	Melting time (s)	Ref.
PSMs	5	123	121	[6]
	100	1248	7	[51]
	10	688	115	[52]
	50	700	500	[53]
	50	2526	45	[54]
	30	392	1142	[55]
	10	596	134	[56]
	5	304	74	[57]
	20	66	446	[58]
	10	745	191	[59]
	100	1368	78	[60]
	10	1796	258	[61]
	150	256	150	[62]
	200	1219	166	[63]
	10	774	240	[64]
	15	323	118	[65]
	20	386	452	[66]
	100	50	120	[67]
	20	609	504	[68]
PSSMs	30	NA	150	[7]
	50	688	NA	[10]
	8	606	NA	[11]
TCMMs	20	682	223	[13]
TAPSS film	10	1186	410	This work

Supplementary Table 6. Parameters of building roof materials for EnergyPlus simulation.

	TAPSS film-applied building	RC ceramic-applied building	PT film-applied building	Normal building
Thickness (mm)	4	2	2	9.5
Thermal conductivity (W/(m·K))	0.34	1.15	0.24	0.16
Density (kg/m^3)	850	2430	660	1121
Thermal absorptance	0.937	0.965	0.940	0.900
Solar absorptance	Cooling mode: 0.296<br>Heating mode: 0.917	0.012	0.969	0.700

Supplementary Table 7. Representative cities and their corresponding climate zones.

Climate zone	Thermal climate zone	Representative city
0A	Extremely hot humid	Porto Velho
0B	Extremely hot dry	Abu Dhabi
1A	Very hot humid	Honolulu
1B	Very hot dry	New Delhi
2A	Hot humid	Hong Kong
2B	Hot dry	Tucson
3A	Warm humid	Melbourne
3B	Warm dry	EI Paso
3C	Warm marine	San Diego
4A	Mixed humid	Beijing
4B	Mixed dry	Albuquerque
4C	Mixed marine	Seattle
5A	Cool humid	Dalian
5B	Cool dry	Denver
5C	Cool marine	Port Angeles
6A	Cold humid)	Shenyang
6B	Cold dry	Great Falls
7	Very cold	Harbin
8	Subarctic/Arctic	Fairbanks

Supplementary Notes

Supplementary Note 1

The photothermal conversion efficiency was defined as the ratio of the heat generated by the photothermal material to the input of solar power, and the calculation formula is as follows:

$$\eta = \frac{Q_{\text{gen}}}{q_{\text{sol}} A} \times 100\%, \tag{S7}$$

where Q_{gen} represents the heat generated by photothermal conversion, q_{sol}=1 kW/m^2 represents the light intensity of simulated sunlight, A represents the surface area of the sample.

When the surface temperature of PT film stabilized, the heat generated by the sample was in balance with the heat dissipation to the ambient environment, which could be described by

$$Q_{\text{gen}} = Q_{\text{loss}} = Q_{\text{conv}} + Q_{\text{rad}}, \tag{S8}$$

where Q_{loss} represents the heat loss to the surroundings, including convective heat loss (Q_{conv}) radiative heat loss (Q_{rad}).

The convection heat loss is calculated by

$$Q_{\text{conv}} = hA\left(T_{\text{sam}} - T_{\text{amb}}\right), \tag{S9}$$

where h represents the convective heat transfer coefficient, T_{sam} represents the equilibrium temperature of the sample, and T_{amb} is ambient temperature. The convective heat transfer coefficient can be calculated by $h=Nu\lambda/l$, where λ represents the thermal conductivity of air, l represents the characteristic length, and Nu is the Nusselt number, which can be calculated by the following equation $Nu=0.54(GrPr)^{0.25}$, where $Gr=g\alpha_V\Delta Tl^3/v^2$ represents the Grashof number, $Pr=v/a$ represents the Prandtl number, α_V represents the expansion coefficient, g represents the gravitational acceleration, ΔT represents the temperature difference between sample and ambient air, v represents the kinematic viscosity, a represents the thermal diffusivity. The values of the thermal properties above were determined by the characteristic temperature $T_m=(T_{\text{sam}}+T_{\text{amb}})/2$ (i.e., the arithmetic average temperature of the boundary layer). For horizontal plate natural convection heat transfer, the characteristic length can be calculated by $l=A/P$, where P represents the perimeter of samples.

The radiative heat loss is calculated by

$$Q_{\text{rad}} = \varepsilon\sigma A\left(T_{\text{sam}}^4 - T_{\text{amb}}^4\right), \tag{S10}$$

where ε represents the emissivity of samples, σ=5.67×10^{-8} W/(m^2·K^4) represents the Stefan-Boltzmann constant.

Supplementary Note 2

According to classical nucleation theory, the energy barrier of homogeneous nucleation (ΔG_{hom}) and heterogeneous nucleation (ΔG_{het}) were defined as[69-72]

$$\Delta G_{\mathrm{hom}} = \frac{16\pi\sigma^3 T_{\mathrm{m}}^2}{3\left(\Delta H_{\mathrm{m}} \Delta T\right)^2}, \tag{S11}$$

$$\Delta G_{\mathrm{het}} = \Delta G_{\mathrm{hom}} f\left(\theta\right), \tag{S12}$$

$$f\left(\theta\right) = \frac{\left(1-\cos\theta\right)^2\left(2+\cos\theta\right)}{4}, \tag{S13}$$

where σ represents water-ice interfacial tension, θ represents water CA, $\Delta T = T_{\mathrm{m}} - T_{\mathrm{c}}$ represents supercooling. Consequently, ice nucleation preferentially occurs at foreign surfaces due to a lower energy barrier (i.e., $\Delta G_{\mathrm{het}} < \Delta G_{\mathrm{hom}}$, $0 < f(\theta) < 1$). For CPCMs, nucleation initiated at the MWCNTs@PDMS interface, while pure PCM nucleated on the aluminum pan surface. The more pronounced ΔT of CPCMs compared to PCM, arises from the larger CA of PCM droplets on the PT-6 surface (Supplementary Fig. 31), which increases $f(\theta)$ and consequently elevates ΔG_{het}.

Supplementary Note 3

As shown in Supplementary Fig. 36, the heat transfer model of the melting ice is given by

$$\Delta Q = Q_{\mathrm{gain,c}} + Q_{\mathrm{gain,r}} - Q_{\mathrm{loss}}, \tag{S14}$$

where ΔQ represents the net heat transferred to the melting ice, $Q_{\mathrm{gain,c}}$ represents the heat conducted from the solid surface to the ice, $Q_{\mathrm{gain,r}}$ represents the heat from the solar radiation, and Q_{loss} represents the heat loss by convection and radiation. Evidently, $Q_{\mathrm{gain,c}}$ is the main heat source for ice melting, which is described by

$$Q_{\mathrm{gain,c}} = Q_{\mathrm{gen}} - Q_{\mathrm{latent}} - Q_{\mathrm{surf}}, \tag{S15}$$

where Q_{gen} represents the heat generated by photothermal effect, Q_{latent} represents the latent heat stored by PCM melting, Q_{surf} represents the heat loss from solid surface by convection and radiation.

Supplementary References

[1] Zhong, H. et al. Plasmonic photothermal superhydrophobic surface with nanotubes thermal insulating blanket for anti-icing and anti-frosting under weak light illumination. *Mater. Today Phys.* **50**, 101625 (2025).

[2] Zhang, C. et al. Hierarchical dandelion-like superhydrophobic surfaces with excellent stability and photothermal performance for efficient anti-/deicing. *Chem. Eng. J.* **510**, 161582 (2025).

[3] Mao M, et al. Scalable robust photothermal superhydrophobic coatings for efficient anti-icing and de-icing in simulated/real environments. *Nat. Commun.* **15**, 9610 (2024).

[4] Wu, S. et al. Superhydrophobic photothermal icephobic surfaces based on candle soot. *Proc. Natl. Acad. Sci.* **117**, 11240-11246 (2020).

[5] Sun, W. et al. Anti-icing and deicing characteristics of photothermal superhydrophobic surfaces based on metal nanoparticles and carbon nanotube materials. *Energy* **286**, 129656 (2024).

[6] Zhang, F., Yan, H. & Chen, M. Multi-scale superhydrophobic surface with excellent stability and solar-thermal performance for highly efficient anti-icing and deicing. *Small* **20**, 2312226 (2024).

[7] Hou, M. et al. Efficient photothermal anti-/deicing enabled by 3D $Cu_{2-x}S$ encapsulated phase change materials mixed superhydrophobic coatings. *Adv. Mater.* **36**, 2310312 (2024).

[8] Sheng, S. et al. Bioinspired solar anti-icing/de-icing surfaces based on phase-change materials. *Sci. China Mater.* **65**, 1369-1376 (2022).

[9] Xie, Z. et al. Photothermal materials with energy-storage properties provide an energy-saving design for highly efficient anti-icing/deicing applications. *Appl. Phys. Lett.* **123**, 4 (2023).

[10] Yang, H. et al. Bio-Inspired Anti-Icing Material as an Energy-Saving Design toward Sustainable Ice Repellency. *Adv. Mater. Technol.* **7**, 2200502 (2022).

[11] Song, L. et al. Multifunctional photothermal phase-change superhydrophobic film with excellent light–thermal conversion and thermal-energy storage capability for anti-icing/de-icing applications. *Langmuir* **38**, 15245-15252 (2022).

[12] Ren, Y. et al. Photothermal superhydrophobic composite coatings based on n-tetradecane@$CaCO_3$/TiN microcapsules for anti-/deicing. *Surf. Coat. Technol.* **485**, 130888 (2024).

[13] Feng, W. et al. All-weather anti-icing and de-icing properties of the carbon-based electro@ photo-thermal material with adaptive temperature control performance. *Prog. Org. Coat.* **188**, 108194 (2024).

[14] Liu, Y. et al. Reversible thermochromic and heat storage coating for adaptive de/anti-icing and thermal regulation. Chem. Eng. J. **482**, 148837 (2024).

[15] Chen, S. et al. Bioinspired metafilms for all-weather energy harvesting: Adaptive thermal regulation and raindrop electricity generation. *Sci. Adv.* **11**, eadu2895 (2025).

[16] Hu, X. et al. Colorful and Temperature-Adaptive Radiative Coolers for All-Season Thermal Management Applications. *Renew. Energy* **242**, 122447 (2025).

[17] Guo, N. et al. Self-Adaptive Colored Radiative Cooling by Tuning Visible Spectra. *Sol. RRL* **7**, 2300512 (2023).

[18] Liu, B. Y. et al. Bioinspired Superhydrophobic All-In-One Coating for Adaptive Thermoregulation. *Adv. Mater.* **36**, 2400745 (2024).

[19] Siddiqui, A. R. et al. One-step fabrication of transparent superhydrophobic surface. *Appl. Surf. Sci.* **542**, 148534 (2021).

[20] Nomeir, B. et al. Durable and transparent superhydrophobic coating with temperature-controlled multi-scale roughness for self-cleaning and anti-icing applications. *Prog. Org. Coat.* **189**, 108338 (2024).

[21] Liu, Y. et al. Eco-friendly fabrication of transparent superhydrophobic coating with excellent mechanical robustness, chemical stability, and long-term outdoor durability. *Langmuir* **38**, 12881-12893 (2022).

[22] Wu, Y., Dong, L., Ran, Q. Facile one-step spraying preparation of fluorine-free transparent superhydrophobic

composite coatings with tunable adhesion for self-cleaning and anti-icing applications. *Appl. Surf. Sci.* **649**, 159193 (2024).
[23] Li, M. et al. Low-cost preparation of durable, transparent, superhydrophobic coatings with excellent environmental stability and self-cleaning function. *Surf. Coat. Technol.* **438**, 128367 (2022).
[24] Luo, W. et al. Fabrication of robust, anti-reflective, transparent superhydrophobic coatings with a micropatterned multilayer structure. *Langmuir* **38**, 7129-7136 (2022).
[25] Liu, M. et al. Transparent superhydrophobic EVA/SiO_2/PTFE/KH-570 coating with good mechanical robustness, chemical stability, self-cleaning effect and anti-icing property fabricated by facile dipping method. *Colloids Surf. A* **658**, 130624 (2023).
[26] Zhou, Y. et al. Temperature-responsive hydrogel with ultra-large solar modulation and high luminous transmission for "smart window" applications. *J. Mater. Chem. A* **2**, 13550-13555 (2014).
[27] Mei, X. et al. A self-adaptive film for passive radiative cooling and solar heating regulation. *J. Mater. Chem. A* **10**, 11092-11100 (2022).
[28] Lin, C. et al. All-weather thermochromic windows for synchronous solar and thermal radiation regulation. *Sci. Adv.* **8**, eabn7359 (2022).
[29] Zhou, Y. et al. Liquid thermo-responsive smart window derived from hydrogel. *Joule* **4**, 2458-2474 (2020).
[30] Fang, Z. et al. Thermal homeostasis enabled by dynamically regulating the passive radiative cooling and solar heating based on a thermochromic hydrogel. *ACS Photonics* **8**, 2781-2790 (2021).
[31] Liang, H. et al. Bio-inspired micropatterned thermochromic hydrogel for concurrent smart solar transmission and rapid visible-light stealth at all-working temperatures. *Light Sci. Appl.* **13**, 202 (2024).
[32] Li, G. et al. Physical crosslinked hydrogel-derived smart windows: anti-freezing and fast thermal responsive performance. *Mater. Horiz.* **10**, 2004-2012 (2023).
[33] Ding, Y. et al. High-transmittance pNIPAm gel smart windows with lower response temperature and stronger solar regulation. *Chem. Eng. J.* **460**, 141572 (2023).
[34] Ding, Y. et al. Low energy consumption thermochromic smart windows with flexibly regulated photothermal gain and radiation cooling. *Appl. Energy* **348**, 121598 (2023).
[35] Li, X. H. et al. Broadband light management with thermochromic hydrogel microparticles for smart windows. *Joule* **3**, 290-302 (2019).
[36] Kong, X. et al. A novel smart window based on co-crosslinked hydrogel with temperature self-adaptability and anti-freezing functions for building energy saving. *Sol. Energy* **284**, 113099 (2024).
[37] Wang, S. et al. A solar/radiative cooling dual-regulation smart window based on shape-morphing kirigami structures. *Mater. Horiz.* **10**, 4243-4250 (2023).
[38] Liu, S. et al. Bioinspired thermochromic transparent hydrogel wood with advanced optical regulation abilities and mechanical properties for windows. Appl. Energy 297, 117207 (2021).
[39] Wang, W. et al. Bidirectional Temperature-Responsive Thermochromic Hydrogels With Adjustable Light Transmission Interval for Smart Windows. *Adv. Funct. Mater.* **34**, 2413102 (2024).
[40] Chen, L. et al. 3D printed hydrogel for soft thermo-responsive smart window. *Int. J. Extrem. Manuf.* **4**, 025302 (2022).
[41] Chen, G. et al. Printable thermochromic hydrogel-based smart window for all-weather building temperature regulation in diverse climates. *Adv. Mater.* **35**, 2211716 (2023).
[42] Wang, K., Zhang, L. & Jiang, X. Freezing-resistant poly (N-isopropylacrylamide)-based hydrogel for thermochromic smart window with solar and thermal radiation regulation. *J. Colloid Interface Sci.* **652**, 663-672 (2023).
[43] Xie, G. et al. Scalable Fabrication of Thermochromic Smart Windows for Broadening Temperature Ranges

and Their Coupled Thermoelectric Power Generation. *ACS Sustain. Chem. Eng.* **12**, 14890-14901 (2024).

[44] Wang, S. et al. Thermochromic smart windows with highly regulated radiative cooling and solar transmission. *Nano Energy* **89**, 106440 (2021).

[45] Yang, Y. S. et al. Temperature-responsive hydroxypropylcellulose based thermochromic material and its smart window application. *RSC Adv.* **6**, 61449-61453 (2016).

[46] Zhang, L. et al. Energy-saving smart windows with HPC/PAA hybrid hydrogels as thermochromic materials. *ACS Appl. Energy Mater*. **4**, 9783-9791 (2021).

[47] Niu, Y. et al. Energy saving and energy generation smart window with active control and antifreezing functions. *Adv. Sci.* **9**, 2105184 (2022).

[48] Xie, L. et al. Engineering Self-Adaptive Multi-Response Thermochromic Hydrogel for Energy-Saving Smart Windows and Wearable Temperature-Sensing. *Small* **19**, 2304321 (2023).

[49] Feng, Y. et al. Entanglement in Smart Hydrogels: Fast Response Time, Anti-Freezing and Anti-Drying. *Adv. Funct. Mater.* **33**, 2211027 (2023).

[50] Lin, Z., Yang, Z., Gao, L. Engineering a polyvinyl butyral hydrogel as a thermochromic interlayer for energy-saving windows. *Mater. Horiz*. **11**, 3127-3142 (2024).

[51] Liu, Q. et al. Robust and Ultra-Efficient Anti-/De-Icing Surface Engineered Through Photo-/Electrothermal Micro-Nanostructures With Switchable Solid-Liquid States. *Adv. Mater.* **37**, 2410941 (2025).

[52] Xie, Z. et al. Heat transfer characteristics of carbon-based photothermal superhydrophobic materials with thermal insulation micropores during anti-icing/deicing. *J. Phys. Chem. Lett.* **13**, 10237-10244 (2022).

[53] Wu, C. et al. Highly efficient solar anti-icing/deicing via a hierarchical structured surface. *Mater. Horiz.* **7**, 2097-2104 (2020).

[54] Ju, G. et al. Photothermal Active-Deicing Nanocoatings with Long-Term Superhydrophobicity Based on In Situ Amorphous Polymers Design. *Small* **21**, 2410484 (2025).

[55] Liu, X. et al. Robust all-waterborne superhydrophobic coating with photothermal deicing and passive anti-icing properties. *ACS Appl. Mater. Interfaces* **15**, 44305-44313 (2023).

[56] Liu, Z., Hu, J. & Jiang, G. Superhydrophobic and photothermal deicing composite coating with self-healing and anti-corrosion for anti-icing applications. *Surf. Coat. Technol.* **444**, 128668 (2022).

[57] Zheng, W. et al. Magnetic responsive and flexible composite superhydrophobic photothermal film for passive anti-icing/active deicing. *Chem. Eng. J.* **427**, 130922 (2022).

[58] Qin, X. et al. An all-weather anti/de-icing coating combining superhydrophobic surfaces with photothermal and electrothermal functions. *J. Mater. Res. Technol.* **35**, 152-163 (2025).

[59] Liu, S. et al. Waxberry-liked micro-nanostructured, superhydrophobic surfaces with enhanced photothermal de-icing and passive anti-icing properties. *Chem. Eng. J.* 503, 158358 (2025).

[60] Yang, X. et al. Ultra-durable photothermal anti-/de-icing superhydrophobic coating with water droplets freezing from the outside in. *J. Colloid Interface Sci.* **682**, 1127-1139 (2025).

[61] Yang, X. et al. Anti/de-icing superhydrophobic coating with durability and self-healing by infiltrating photothermal self-stratifying organic layers into plasma-sprayed porous Al_2O_3–13% TiO_2 underlayer. *Surf. Interfaces* **54**, 105305 (2024).

[62] Zhang, J. et al. A fluorinated MXene-doped superhydrophobic coating with mechanochemical robustness, repairable wettability and photothermal conversion for highly efficient anti/de-icing. *Chem. Eng. J.* **498**, 155499 (2024).

[63] Yang, X. et al. Robust photothermal anti/de-icing hydrophobic coating based on polydopamine (PDA) composition. *Appl. Surf. Sci.* **681**, 161415 (2025).

[64] Xie, Z. et al. Photothermal trap with multi-scale micro-nano hierarchical structure enhances light absorption

and promote photothermal anti-icing/deicing. *Chem. Eng. J.* **435**, 135025 (2022).

[65] Liu, L. et al. Anti-/Deicing Membranes with Damage Detection and Fast Healing. *Adv. Funct. Mater.* 34, 2404760 (2024).

[66] Zhou, X. et al. Robust superhydrophobic coating for photothermal anti-icing and de-icing via electrostatic powder spraying. *Prog. Org. Coat.* **197**, 108778 (2024).

[67] Zhao, X. et al. A Superhydrophobic and Recyclable Coating with Strong Robustness for Anti-Icing Applications. *Adv. Mater. Technol.* **10**, 2401929 (2025).

[68] Liu, Z. et al. Construction of multilayer graphene-based photothermal superhydrophobic coatings: synergistic de-icing mechanism and investigation of multiscale heat transfer properties. *Carbon* **242**, 120401 (2025).

[69] Schutzius, T. M. et al. Physics of icing and rational design of surfaces with extraordinary icephobicity. *Langmuir* **31**, 4807-4821 (2015).

[70] Zhang, Z. & Liu, X. Y. Control of ice nucleation: freezing and antifreeze strategies. *Chem. Soc. Rev.* **47**, 7116-7139 (2018).

[71] Fletcher, N. H. Size effect in heterogeneous nucleation. *J. Chem. Phys.* **29**, 572-576 (1958).

[72] He, H. & Guo, Z. Superhydrophobic materials used for anti-icing: Theory, application, and development. *iScience* **24**, 103357 (2021).
