# Supplementary material for: A self-regulated photothermal anti-/deicing film for all-season applications
Source: Nat Commun. 2026 Feb 11;17:2632. doi: 10.1038/s41467-026-69494-x (PMC13004981; doi:10.1038/s41467-026-69494-x)
Supplement: Supplementary file 2 — Description of Additional Supplementary File [file 41467_2026_69494_MOESM2_ESM.pdf]

## The Description of Additional Supplementary Files

**Supplementary Movie 1:** 10 ⍰L droplets sliding along the MNTS film with a tilt angle of 1.5° (1/8× speed)

**Supplementary Movie 2:** Impact of a 10 ⍰L water droplet on the MNTS film with an impact velocity of 0.5 m/s (1/40× speed)

**Supplementary Movie 3:** Self-cleaning test on a MNTS-coated glass slide (2× speed)

**Supplementary Movie 4:** Wettability of MNTS-coated glass slide before and after had rubbing test (2× speed)

**Supplementary Movie 5:** Wettability of MNTS-coated glass slide after water jet impact test for 15 s (2× speed)

**Supplementary Movie 6:** Heating and cooling cycles of a PNDE hydrogel in water baths at 20℃ and 40℃ (4× speed)

**Supplementary Movie 7:** Icing process of a 10 ⍰L water droplet on a PTPCC film under -20℃and 20%RH (6× speed)

**Supplementary Movie 8:** Icing process of a 10 ⍰L water droplet on a TAPSS film under -20℃ and 20%RH (10× speed)

**Supplementary Movie 9:** Dynamic process of a 10 ⍰L water droplet impact on a with 3° inclined TAPSS film under -20℃ and 20%RH (1/25× speed)

**Supplementary Movie 10:** Deicing process of a 10 µL water droplet on a TAPSS film under 20℃ and 1 sun illumination (10× speed)

**Supplementary Movie 11:** Defrosting process on a aircraft model under -15℃, 80%RH, 1 sun illumination (10× speed) Supplementary Movie 12: Defrosting process on a wind turbine model under -15℃, 80%RH, 1 sun illumination (60× speed)
