# Supplementary material for: A self-regulated photothermal anti-/deicing film for all-season applications
Source: Nat Commun. 2026 Feb 11;17:2632. doi: 10.1038/s41467-026-69494-x (PMC13004981; doi:10.1038/s41467-026-69494-x)
Supplement: Supplementary file 15 — Reporting Summary [file 41467_2026_69494_MOESM15_ESM.pdf]

nature portfolio

Corresponding author(s): Chi Yan Tso and Fuqiang Chu

Last updated by author(s): Dec 19, 2025

Reporting Summary

Nature Portfolio wishes to improve the reproducibility of the work that we publish. This form provides structure for consistency and transparency in reporting. For further information on Nature Portfolio policies, see our Editorial Policies and the Editorial Policy Checklist.

Statistics

For all statistical analyses, confirm that the following items are present in the figure legend, table legend, main text, or Methods section.

n/a	Confirmed	
☐	☒	The exact sample size (*n*) for each experimental group/condition, given as a discrete number and unit of measurement
☐	☒	A statement on whether measurements were taken from distinct samples or whether the same sample was measured repeatedly
☒	☐	The statistical test(s) used AND whether they are one- or two-sided<br>*Only common tests should be described solely by name; describe more complex techniques in the Methods section.*
☒	☐	A description of all covariates tested
☒	☐	A description of any assumptions or corrections, such as tests of normality and adjustment for multiple comparisons
☐	☒	A full description of the statistical parameters including central tendency (e.g. means) or other basic estimates (e.g. regression coefficient) AND variation (e.g. standard deviation) or associated estimates of uncertainty (e.g. confidence intervals)
☒	☐	For null hypothesis testing, the test statistic (e.g. F, t, r) with confidence intervals, effect sizes, degrees of freedom and P value noted<br>*Give P values as exact values whenever suitable.*
☒	☐	For Bayesian analysis, information on the choice of priors and Markov chain Monte Carlo settings
☒	☐	For hierarchical and complex designs, identification of the appropriate level for tests and full reporting of outcomes
☒	☐	Estimates of effect sizes (e.g. Cohen's d, Pearson's r), indicating how they were calculated

Our web collection on statistics for biologists contains articles on many of the points above.

Software and code

Policy information about availability of computer code

Data collection	To predict the energy saving potential, we have employed an open source software, EnergyPlus (version: 22.1.0) to calculate the energy consumption.
Data analysis	We have used Thermo Scientific™Avantage (version:5.9931) for analyzing XPS spectra.

For manuscripts utilizing custom algorithms or software that are central to the research but not yet described in published literature, software must be made available to editors and reviewers. We strongly encourage code deposition in a community repository (e.g. GitHub). See the Nature Portfolio guidelines for submitting code & software for further information.

Data

Policy information about availability of data

All manuscripts must include a data availability statement. This statement should provide the following information, where applicable:

- Accession codes, unique identifiers, or web links for publicly available datasets
- A description of any restrictions on data availability
- For clinical datasets or third party data, please ensure that the statement adheres to our policy

The data that supports the findings of this study are available from the corresponding authors upon request.

Research involving human participants, their data, or biological material

Policy information about studies with human participants or human data. See also policy information about sex, gender (identity/presentation), and sexual orientation and race, ethnicity and racism.

Reporting on sex and gender	N/A
Reporting on race, ethnicity, or other socially relevant groupings	N/A
Population characteristics	N/A
Recruitment	N/A
Ethics oversight	N/A

Note that full information on the approval of the study protocol must also be provided in the manuscript.

Field-specific reporting

Please select the one below that is the best fit for your research. If you are not sure, read the appropriate sections before making your selection.

☐ Life sciences ☐ Behavioural & social sciences ☒ Ecological, evolutionary & environmental sciences

For a reference copy of the document with all sections, see nature.com/documents/nr-reporting-summary-flat.pdf

Ecological, evolutionary & environmental sciences study design

All studies must disclose on these points even when the disclosure is negative.

Study description	We develop a temperature-adaptive photothermal storage superhydrophobic (TAPSS) film that dynamically switches between black (cold conditions) and white (hot environments). The TAPSS film synergizes outstanding solar modulation, superhydrophobicity and phase-change energy storage, enabling summer overheating prevention and winter anti-/deicing. Moreover, it demonstrates excellent scalability, flexibility, energy-saving potential and durability, positioning it as a promising next-generation anti-/deicing solution.
Research sample	N/A
Sampling strategy	N/A
Data collection	For the optical properties, the data was collected by commercial spectrometers. For photothermal test and cooling field test, the data was collected by commercial data logger. For surface wettability, the data was collected by a high speed camera. All these experiments and data collection was conducted by Dr. Jiayu Du. Detailed procedure can be found in Methods section and Supplementary Information.
Timing and spatial scale	For field test, the data was collected in in Hong Kong, china (June 6-10, 2025) during clear sunny days. The temperature was recorded in every 10 s.
Data exclusions	No data was excluded.
Reproducibility	For each measurement of the optical performance, surface wettability, and photothermal performance, the sample was measured at least 3 times at the same procedure and be averaged to verify the reproducibility. All attempts to repeat the experiment were successful.
Randomization	This is not relevant to our study as all the samples were designed/fabricated/characterized under standard procedures or common routes.
Blinding	This is not relevant to our study because no participant is required for obtaining the results.

Did the study involve field work? ☒ Yes ☐ No

Field work, collection and transport

Field conditions	In Hong Kong, we have selected the rooftop with minimized shading to carry out the filed test. The field test was conducted on sunny days. The environmental parameters (ambient temperature, humidity, sunlight) have been given in the paper.

Location	Hong Kong (22.34 North, 114.17 East)
Access & import/export	N/A
Disturbance	N/A

Reporting for specific materials, systems and methods

We require information from authors about some types of materials, experimental systems and methods used in many studies. Here, indicate whether each material, system or method listed is relevant to your study. If you are not sure if a list item applies to your research, read the appropriate section before selecting a response.

Materials & experimental systems

n/a	Involved in the study
☒	☐ Antibodies
☒	☐ Eukaryotic cell lines
☒	☐ Palaeontology and archaeology
☒	☐ Animals and other organisms
☒	☐ Clinical data
☒	☐ Dual use research of concern
☒	☐ Plants

Methods

n/a	Involved in the study
☒	☐ ChIP-seq
☒	☐ Flow cytometry
☒	☐ MRI-based neuroimaging

Plants

Seed stocks	N/A
Novel plant genotypes	N/A
Authentication	N/A
